# The persistent influence of caste on under-five mortality: Factors that explain the caste-based gap in high focus Indian states

**DOI:** 10.1371/journal.pone.0211086

**Published:** 2019-08-20

**Authors:** Jayanta Kumar Bora, Rajesh Raushan, Wolfgang Lutz

**Affiliations:** 1 Wittgenstein Centre for Demography and Global Human Capital (IIASA, VID/ ÖAW and WU), Austria; 2 International Institute for Applied Systems Analysis, Laxenburg, Austria; 3 Indian Institute of Dalit Studies, New Delhi, India; Institute of Economic Growth, INDIA

## Abstract

**Objective:**

Although under-five mortality rate (U5MR) is declining in India, it is still high in a few selected states and among the scheduled caste (SC) and scheduled tribe (ST) population of the country. This study re-examines the association between caste and under-five deaths in high focus Indian states following the implementation of the country’s National Rural Health Mission (NRHM) program. In addition, we aim to quantify the contribution of socioeconomic determinants in explaining the gap in under-five death risk between the SC/ST population and non-SC/ST population in high focus states in India.

**Data and method:**

Using data from the National Family Health Survey (NFHS), we calculated the U5MR by applying a synthetic cohort probability approach. We applied a binary logistic regression model to examine the association of under-five deaths with the selected covariates. Further, we used Fairlie's decomposition technique to understand the relative contribution of socioeconomic variables on under-five death risk between the caste groups.

**Findings:**

In high focus Indian states, the under-five mortality risk between well-off and deprived caste children has declined in the post-NRHM period, indicating a positive impact in terms of reducing caste-based inequalities in the high focus states. Despite the reduction in under-five death risk, children belonging to the SC population experience higher mortality rates than children belonging to the non-SC/ST population from 1992 to 2016. Both macro level (district level mortality rates) and individual (regression analysis) analyses showed that children belonging to SCs experience the highest likelihood of dying before their fifth birthday. A decomposition analysis revealed that 83% of the caste-based gap in the under-five deaths is due to the distribution of women’s level of educational attainment and household wealth between the SC/ST and non-SC/ST population. Program indicators such as place of birth and number of antenatal care (ANC) visit also contributed significantly to widening caste-based gaps in U5MR.

**Conclusion:**

The study indicates that there is still room to improve access to health facilities for mothers and children belonging to deprived caste groups in India. Continuous efforts to raise the level of maternal education and the economic status of people belonging to deprived caste groups should be pursued simultaneously.

## Introduction

Mortality among children of age five and below has declined in most countries, with the decline accelerating since mid-2000 [1,2]. In the era of the Sustainable Development Goals (SDGs), the issue continues to be a major public health concern, particularly in low-and middle-income countries (LMICs). India, one of the important LMICs, has experienced a faster decline in U5MR since the inaugural of National Rural Health Mission in 2005. Despite the reduction of the U5MR, India contributes the highest number of deaths in children under five. There exist large disparities in the U5MR across regions and the social groups in India.

The burden of child mortality in high focus states of India has been the concern of policymakers and researchers equally. The high-focus states in India were designated as such by the Indian government because of their persistently high child mortality and relatively poor socio-economic and health indicators. A recent study demonstrated that the majority of the districts in the high focus states are not likely to achieve the SDGs concerning the preventable death of newborns [3]. Within the high focus states, the socially disadvantaged population groups carry a higher burden of under-five deaths. This paper re-examines the disadvantage in mortality rate experienced by the Scheduled Caste (SC) and Scheduled Tribe (ST) children in high focus states using the most recent data. It also aims to quantify the relative contribution of socioeconomic determinants to under-five death risk by explaining the gap between socially disadvantaged and non-disadvantaged castes in high focus states. Identifying disadvantaged groups in high focus states can help to reduce the absolute and relative burden of under-five deaths in India.

### Caste affiliation and under-five mortality in India

The Indian caste system is a traditional system of social stratification that has existed for more than three thousand years [4]. It is a social stratification system of self-governing and closed groups or communities called Jatis. These Jatis are assigned by birth and remains the same throughout an individual’s life. The ancient Varna system divided Hindu society into initially four, and later five, distinct Varna or castes, that are mutually exclusive, hereditary, endogamous and occupation-specific. These are the Brahmins (priests), Kshatriyas (warriors), Vaisyas (traders and merchants) and Sudras (those engaged in menial jobs) and those doing the most despicable menial jobs- the Ati Sudras or the former untouchables [5]. The ‘untouchables’ have the lowest social standing. Another way of categorizing the castes, which is now used in India to direct specific policies, is SCs, STs, other backward classes (all disadvantaged groups) and general castes (non-disadvantaged castes). People who belong to SCs were previously referred to as “untouchables,” while the STs are communities of people living in tribal areas (mainly forest). SCs and STs are historically marginalized and disadvantaged social groups and are officially recognized and listed by the Indian Constitution. Interestingly, although castes originated within the Hindu religion, it also exists in the other religious groups in India, such as Islam and Christian [6]. According to data obtained from the 2011 census of India, together, they constitute 25.2% of the country’s total population (with SCs contributing 16.6% and STs 8.6%).

Despite continuing efforts by modern governments to redress the effects of the caste system through a system of reservation (positive discrimination), caste remains a significant line of social division in India. The Indian Constitution has given people belonging to disadvantaged groups a special status since 1950 and makes provision for quotas in politics, education and job opportunities, as well as various other arrangements, including laws to abolish practices prolonging social inequities and development programs specially designed to cater to the needs of these groups [7]. However, they continue to face multiple disadvantages compared to the rest of the population [8–10] and still have lower socioeconomic development indicators than the rest of the population [11]. These people are generally exposed to poor living conditions, observe a poor diet, and have limited access to health care. In addition to their low socioeconomic circumstances, people in disadvantaged castes experience other adverse circumstances such as caste-based discrimination while accessing the health care system in India [12]. Besides, their life expectancy is relatively low, and both child and adult mortality are relatively high [13]. People belonging to disadvantaged castes constitute almost 50% of all maternal deaths in the country, and their children are more undernourished compared to the rest of the population [14,15].

The association between caste and child mortality is well documented [13,16–24]. In general, previous studies showed that children belonging to disadvantaged castes such as SCs/STs experience a higher likelihood of death compared to children belonging to non-deprived castes. It has also been found that caste differences in infant and child mortality are substantially reduced when parental socioeconomic characteristics are held constant [21]. In a recent study by Ranjan et al. (2016) [25], the authors concluded that the gap in infant mortality between tribal and non-tribal populations was substantial in the early months after birth, narrowed between the fourth and eighth months, and grew after that. The study by Dommaraju and colleagues (2008) [17] examined the association of caste on child mortality and maternal health care utilization in rural India. They concluded that children belonging to lower castes have a higher risk of death and that women belonging to the lower castes have lower rates of antenatal and delivery care utilization than children and women belonging to the upper castes. The study further suggested the need to target low-caste members in the provision of maternal and child health services.

Has the association between caste and under-five mortality been fading away in recent years due to the government’s health programs? In 2005, the Indian government launched the NRHM, renamed as the National Health Mission (NHM) in 2013, to improve the availability of and access to quality health care for the women and children, especially in rural areas. The program was introduced with a special focus on the nine socioeconomically disadvantaged high focus states. In the same period, the reproductive, maternal, newborn, child, and adolescent health (RMNCH+A) approach was launched to address the delays in accessing and utilizing health care and services by mothers and children. Many components of the NHM directly address the issues related to health and death status of children under age five. There have been numerous initiatives under the NRHM-NHM to enhance newborn care and delivery. Janani Suraksha Yojana (JSY), a safe motherhood scheme, was introduced to reduce maternal and infant mortality by promoting institutional delivery among pregnant women by providing conditional cash assistance. The broader institutional framework of NHM complements the JSY cash incentive by providing comprehensive healthcare, including antenatal and post-natal services, transport to facilities, and support services from the Accredited Social Health Activists (ASHA). It includes several support services administered by community health workers to encourage pregnant women to use healthcare facilities for childbirth, along with at least three antenatal check-ups [26].

Following the implementation of NRHM, India has avoided nearly one million children deaths across socioeconomic groups between 2005 and 2015 [27]. The program is credited with substantially reduced the inequities in maternal health services through increased institutional delivery and antenatal care in the high focus and deprived states of India [28].

Does this substantial coverage of maternal and health care services in recent years, reduce the caste-based gaps in U5MR in high focus states? Recently available nationally representative data, commonly known as the NFHS allows us to re-examine the association between caste and child mortality in India. To our knowledge, this question had not previously been answered in recent literature. In the present study, we aimed to extend the previous knowledge on caste disparity in several directions. First, we re-examined the association between caste and under-five death in the high focus states in the most recent times. Our study is exclusively based on high focus states in north-central and eastern India, which contributed nearly 46% of the total number of under-five deaths between 2005 and 2015 and were found to be lagging behind in terms of achieving the SDG goals on U5MR [3]. Secondly, we provide district-level estimates of U5MR for the SC and ST population. To our knowledge, no previous study has examined district level variations in the U5MR among disadvantaged castes. Finally, we explain the caste-based gap in under-five death risk by applying an extension Oaxaca-type decomposition for nonlinear models, as suggested by Fairlie [29]. The findings of this study can help to understand the factors behind the pervasive gap in U5MR between deprived and other caste groups in India.

## Data

### Ethics statement

The study is based on an anonymous publicly available dataset with no identifiable information on the survey participants and no ethics statement is required for this research work.

We used data from the fourth round of the Indian Demographic Health Surveys (DHS), commonly known as NFHS conducted between 2015 and 2016. Data from the previous three rounds of NFHS were only used for trend analysis. The 2015–2016 NFHS survey was conducted by the International Institute of Population Sciences (IIPS), Mumbai under the stewardship of the Ministry of Health and Family Welfare (MoHFW) of the government of India. The survey was based on 1,315,617 births from 601,509 households and was covered by 699,686 interviews with women aged between 15 and 49 years old. The sample was selected using a two-stage sample design and covers all 640 districts as per the 2011 census. During the first stage, villages were selected as the primary sampling units (PSUs) for rural areas with probability proportional to size, while for urban areas, census enumeration blocks (CEB) were used. During the second stage, a random selection of 22 households in each PSU and CEB was made for rural and urban areas, respectively. The unit level data is available from the DHS data repository and can be accessed on request. A detailed description of the survey design of the NFHS-4 is available in the national report [30].

We restricted our analysis to the high focus states as done in previous studies [31–34] with women belonging to 304 districts according to the 2011 census of India. The nine high focus states are Assam, Bihar, Chhattisgarh, Jharkhand, Madhya Pradesh, Odisha, Rajasthan, Uttarakhand, and Uttar Pradesh. These are populous states, containing 48.5% of India's population. They are located in the north-central and eastern belt of the country, are characterized by poor demographic and health indicators and have under-five and neonatal mortality rates higher than the national level.

### Methods and measures

#### Outcome variable

The outcome variable in the study is the surviving status of children under age five. We assigned a value of “1” if the child died and “0” if the child was alive for the outcome variable.

#### Predictors

The predictors used in this analysis are broadly divided into three categories: demographic, socioeconomic, and program variables. These variables were considered as they were found to be important determinants of child mortality in previous literature. Under demographic variables, we included the sex of the child, the mother’s age at the birth of her first child (<20, 20–24 and 25 years or more) and the birth order of the children (1, 2–3 and 4+).

Under socioeconomic variables, we included caste affiliation, mothers level of educational attainment, type of residence and wealth quintile of the household, type of fuel used for cooking, type of toilet facilities and source of drinking water. The caste group is the core predictor used in this analysis. We categorized caste into three categories, namely SC, ST, and non-SCST. We categorized the mother level of education into four groups: no education, primary (1 to 5 years of schooling), secondary (6 to 12 years of schooling) and higher (more than 12 years of education).

In terms of wealth, the NFHS survey did not collect information on income. The economic status of a woman was assessed by computing a composite index of household wealth, indicating possession of wealth or assets by the household to which they belonged. We computed the wealth quintile of the household separately using the methodology followed in the fourth round of the NFHS. We excluded the variables of sanitation, the source of drinking water, and cooking fuel while constructing the wealth index. Using the total score, a household was categorized as belonging to the poorest, poorer, middle, richer, and richest group. Among the program indicators, the place of birth of the child (home vs. institutional delivery) and frequency of antenatal care (ANC) visits (none, 1 time, 2–3 times and 4+) during the pregnancy of last birth was considered in our analysis.

### Measures

#### U5MR estimation

Mortality estimation was carried out using the individual-level data on child birth histories that information gathered from women aged 15 to 49 years surveyed in the NFHS. In the analysis, we considered only those births and deaths that took place five years preceding the survey. However, we chose the reference period of the district level U5MR estimates as “ten years prior to the survey date” in order to maximize the size of the sample needed to estimate those rates at the district level. We applied direct methods of mortality-rate estimation using data on the children's date-of-birth and their survival status, as well as the date-of-death and age at death of deceased children. Rutstein and Rojas describe the synthetic cohort probability approach using the full birth history data of women aged 15 to 49 to estimate the U5MR rate [35] on DHS data. A synthetic cohort life table approach used in a previous study [3]. This approach is the same method that was used to produce the child mortality rate in DHS reports using the STATA package “syncmrates” [36].

We carried out the district level estimations of the U5MR for SCs, STs, and non-SC/ST populations separately, with a 95% confidence interval and level of significance. Our inferences regarding the U5MR are strictly based on the districts for which 1) estimated U5MR are statistically significant at a 10% level of significance, and 2) estimated mortality rates are higher than zero. Thus, we provide all estimates in the appendices to this paper, and showed significant, insignificant, and “not enough sample” in the districts for estimates separately.

#### Statistical analysis

We used bivariate analysis to examine the differences in outcome and selected predictors between the SC, ST, and non-SC/ST population. A binary logistic regression model was employed to examine the association between under-five deaths and exposure variables. All exposure variables were tested with the variance inflation factor (VIF) to account for possible multi-collinearity before using the binary logistic regression model.

#### Decomposition analysis

The Blinder–Oaxaca decomposition technique [37,38] is commonly used to identify and quantify the factors associated with inter-group differences in the mean level of outcome. This technique, however, is not appropriate if the outcome variable is binary, such as child mortality. Hence, we used the extension of the Blinder–Oaxaca technique developed by Fairlie [29] which is appropriate for binary models, to decompose the gap between social groups with under-five death risk into contributions that can be attributed to different factors. For the decomposition analysis, we used the *‘fairlie’* command available for Stata. A detailed description of this method is discussed in the appendix (S1 Appendix). We used STATA S.E. 15.0 (STATA Corp., Inc., College Station, TX) version software to carry out the mentioned analysis in this study.

## Results

### Level and trends in U5MR among different social groups in high focus states of India

**Fig 1:**
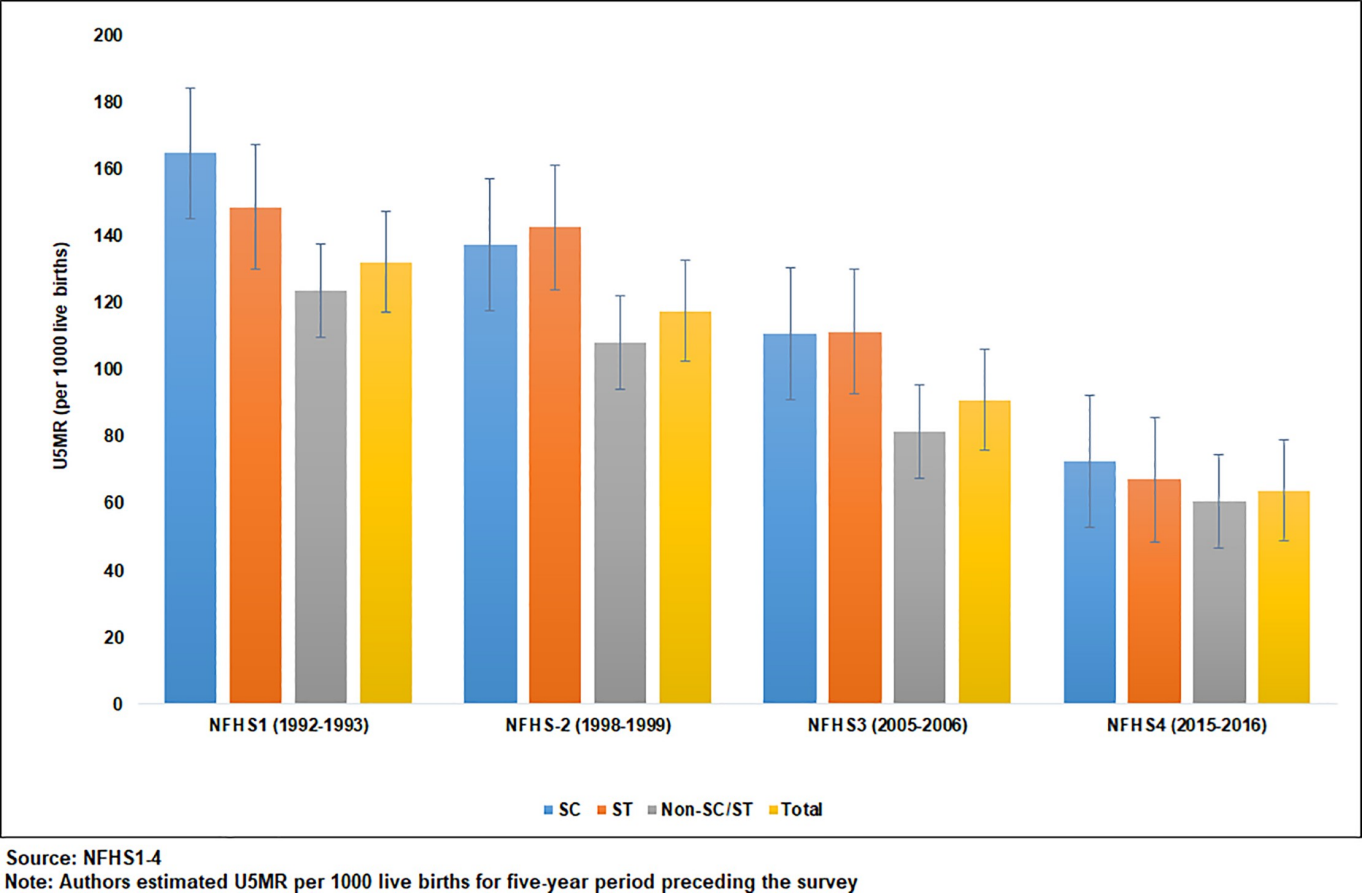
Trends in U5MR among social groups in high focus states, India 1992–2016.

Fig 1 shows the trends in U5MR within the SC, ST, and non-SC/ST populations in high focus states together for 1992–93 to 2015–16. It is observed that despite U5MR having declined the most among children belonging to STs, it is still higher among SCs and STs than among the non-SC/ST population from 1992 to 2016. Compared to 1992–2005, the reduction in the U5MR was higher in the most recent period irrespective of caste groups. It is also clear from Fig 1 that the U5MR in high focus states is substantially higher than the target specified in SDG3 (at least as low as 25 per 1000 live births in 2030) and the national average (50 deaths per 1000 live births in NFHS4) for preventable deaths among children under five. On average, the estimated U5MR of high focus states are more than double the amount specified in the SDG3 target across all caste groups.

Table 1 shows the current level of U5MR of SC, ST, and non-SC/ST population in the selected high focus states. The estimation result is presented with *p-*value and a 95% confidence interval (CI). The results reveal that U5MR for SC children belonging to Bihar, Jharkhand, Madhya Pradesh, Rajasthan, and Uttar Pradesh are substantially higher than that of Non-SC/ST children. On the other hand, U5MR of ST children belonging to Chhattisgarh, Jharkhand, Madhya Pradesh, Odisha, and Rajasthan is relatively higher than the corresponding U5MR of non-SC/ST population.

**Table 1:** U5MR (per 1000 live births) in five years preceding the survey for SC,ST and Non-SC/ST population in high focus states of India, 2015–16.

High focus states	SC			ST			Non-SC/ST		
	U5MR	95% CI		U5MR	95% CI		U5MR	95% CI	
		Lower	Upper		Lower	Upper		Lower	Upper
Assam	50.3	31.9	68.7	51.0	39.3	62.7	58.1	52.9	63.4
Bihar	72.9	65.0	80.9	52.5	38.7	66.2	54.0	50.2	57.8
Chhattisgarh	56.4	40.1	72.8	80.0	69.5	90.6	56.5	47.0	65.9
Jharkhand	59.5	47.7	71.2	63.8	55.2	72.4	48.6	42.0	55.2
Madhya Pradesh	68.5	62.4	74.6	78.3	71.5	85.0	57.8	53.4	62.2
Odisha	45.3	35.4	55.1	65.1	53.8	76.5	39.7	33.9	45.4
Rajasthan	61.8	54.0	69.5	57.8	50.2	65.5	45.3	41.1	49.4
Uttar Pradesh	85.5	79.9	91.1	58.5	37.0	80.1	76.0	72.5	79.4
Uttarakhand	43.8	32.9	54.7	35.9	11.8	60.0	48.0	41.3	54.7
**India**	**55.8**	**53.7**	**58.0**	**57.2**	**53.7**	**60.8**	**46.6**	**45.4**	**47.9**

### District level variation in U5MR by caste group in the districts of high focus states

**Fig 2:**
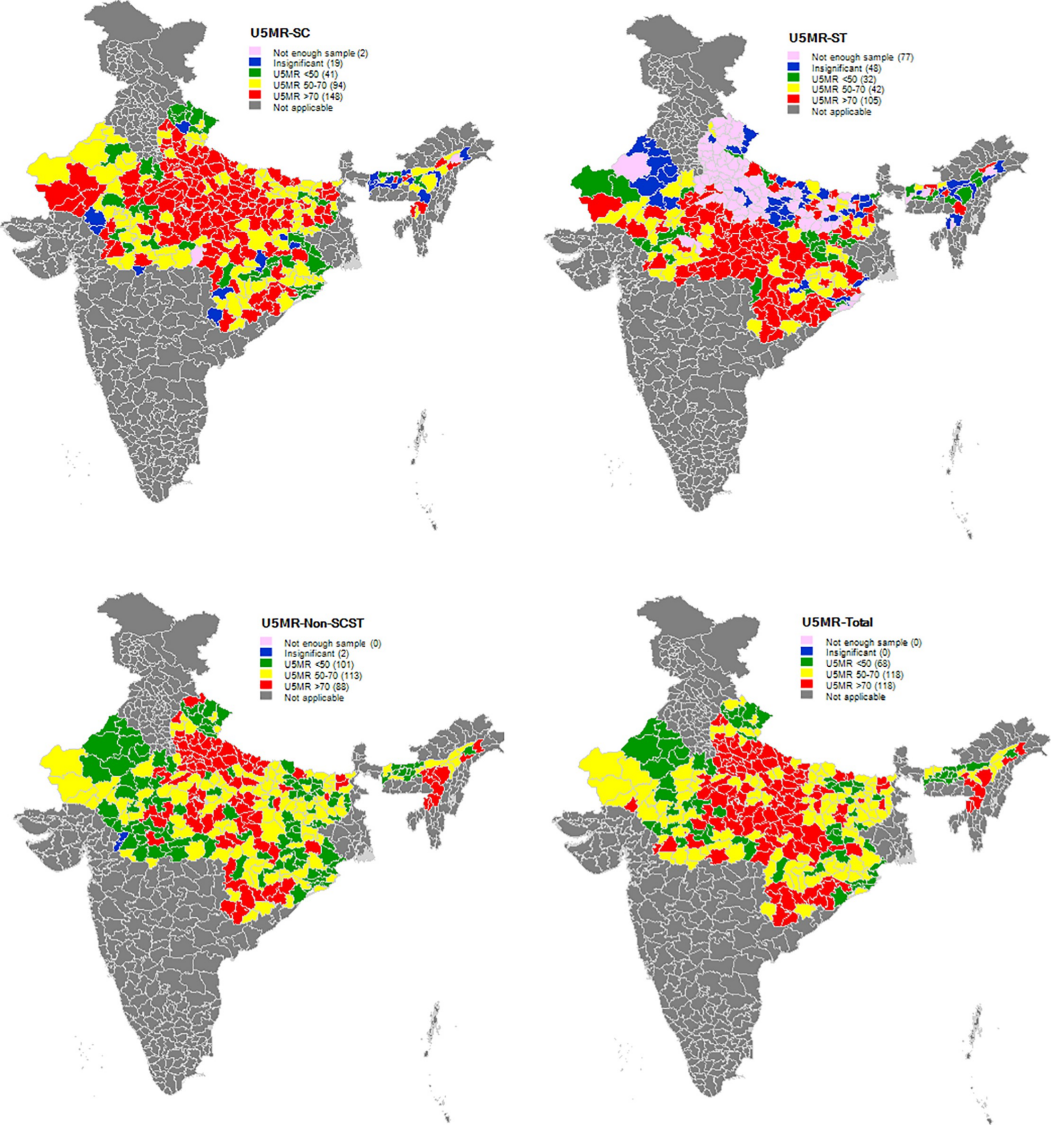
District wise U5MR (per 1000 live births) for SC, ST, non-SC/ST and total populations in high focus states of India, 2015–16.

We present district-level estimates of U5MR (S1 Table and Fig 2) for the SC, ST, non-SC/ST, and total population separately. Each estimated value is supported by a *p-*value and a 95% confidence interval to indicate the statistical significance of the U5MR estimate. Fig 2 presents district-level estimates of U5MR by caste groups. While 59% (105 of 179 districts with significant estimates) of the districts have relatively higher mortality for the ST population (higher than 70 per 1000 live births), only 29% (88 of 302 districts with significant estimates) of districts have higher mortality for the non-SC/ST population. The corresponding figure for the SC population is 52% (148 of 283 districts with significant estimates). On the other hand, 14% (41 out of 283) of SCs, 18% (32 out of 179) of STs and 33% (101 out of 302) of non-SC/STs belong to districts with a U5MR range of below 50. Our findings show that poor performing districts in U5MR for the SC population are geographically concentrated in the states of Rajasthan, Uttar Pradesh, Bihar, and Madhya Pradesh, whereas for STs populations, they are concentrated in Madhya Pradesh, Chhattisgarh, and Odisha.

### Socioeconomic differentials of each caste group

Table 2 shows differences in the selected demographic, socioeconomic, and program indicators from SCs, STs, and the non-SC/ST population. Under-five deaths is higher among SCs and STs compared to the non-SC/ST group. While the mothers of approximately 40% of the SC and ST children have their first child before the age of 20 years, only 35% of the mothers of non-SC/ST children gave birth for the first time before age 20. There are more children with birth order of four or higher among the SC (25%) and ST (21%) population compared to that for non-SC/ST children (19%). We also observed that the mother’s level of education varies widely by caste group. The number of mothers with a secondary and higher level of educational attainment is lower, and no education is higher for the mothers of SC/ST children than for the mothers of non-SC/ST children. About 66% of SC and 82% of ST children belong to poorest and poorer households compared to only 48% of non-SC/ST children. It is worth to mention here that the level of poverty in the high focus states is higher than that at national aggregate level. Similarly, about 82% of SC and 92% of ST children live in rural areas, whereas 76% of the non-SC/ST children are rural inhabitants.

**Table 2:** Comparison of selected characteristics of children under five by social groups of high focus states in India, 2015–16.

Characteristics	SC		ST		Non SC/ST		Total	
	N	%	N	%	N	%	N	%
**Under-five mortality**								
No	20,800	95.7	17,028	96.4	71,437	96.7	109,265	96.4
Yes	943	4.3	641	3.6	2,476	3.3	4,060	3.6
**Sex of the child**								
Female	9,923	45.6	8,250	46.7	33,080	44.8	51,253	45.2
Male	11,820	54.4	9,419	53.3	40,833	55.2	62,072	54.8
**Mother’s age at first birth (yr)**								
<20	8,744	40.2	6,968	39.4	25,540	34.6	41,252	36.4
20–24	10,706	49.2	8,399	47.5	37,974	51.4	57,079	50.4
≥ 25	2,293	10.5	2,302	13.0	10,399	14.1	14,994	13.2
**Birth Order**								
First	6,484	29.8	6,159	34.9	25,682	34.7	38,325	33.8
2–3	9,769	44.9	7,742	43.8	34,249	46.3	51,760	45.7
4+	5,490	25.2	3,768	21.3	13,982	18.9	23,240	20.5
**Mother’s level of education**								
No education	9,249	42.5	8,631	48.8	23,750	32.1	41,630	36.7
Primary	3,640	16.7	2,755	15.6	10,236	13.8	16,631	14.7
Secondary	7,604	35.0	5,715	32.3	31,762	43.0	45,081	39.8
Higher	1,250	5.7	568	3.2	8,165	11.0	9,983	8.8
**Type of residence**								
Urban	3,859	17.7	1,484	8.4	18,089	24.5	23,432	20.7
Rural	17,884	82.3	16,185	91.6	55,824	75.5	89,893	79.3
**Wealth quintile (household)**								
Poorest	9,009	41.4	10,424	59.0	19,722	26.7	39,155	34.6
Poorer	5,308	24.4	3,973	22.5	15,631	21.1	24,912	22.0
Middle	3,500	16.1	1,712	9.7	13,167	17.8	18,379	16.2
Richer	2,409	11.1	999	5.7	12,378	16.7	15,786	13.9
Richest	1,517	7.0	561	3.2	13,015	17.6	15,093	13.3
**Type of fuel used for cooking**^**&**^								
Clean fuel & food not cooked	3,746	17.2	1,253	7.1	21,592	29.2	26,591	23.5
Solid fuel	17,997	82.8	16,416	92.9	52,321	70.8	86,734	76.5
**Type of toilet**^**&**^								
Improved toilet	6,451	29.7	3,442	19.5	36,267	49.1	46,160	40.7
Not improved toilet	15,292	70.3	14,227	80.5	37,646	50.9	67,165	59.3
**Source of drinking water**^**&**^								
Improved water	19,931	91.7	13,838	78.3	67,931	91.9	101,700	89.7
Not improved water	1,812	8.3	3,831	21.7	5,982	8.1	11,625	10.3
**Place of birth**								
Home delivery	5,787	26.6	5,919	33.5	16,456	22.3	28,162	24.9
Institutional delivery	15,956	73.4	11,750	66.5	57,457	77.7	85,163	75.1
**Antenatal visits during pregnancy (last birth)**								
None	5,451	25.1	3,924	22.2	14,872	20.1	24,247	21.4
1 visit	1,747	8.0	969	5.5	5,345	7.2	8,061	7.1
2–3 visits	8,199	37.7	6,293	35.6	26,953	36.5	41,445	36.6
4+ visits	6,346	29.2	6,483	36.7	26,743	36.2	39,572	34.9
**N**	**21,743**	**100.0**	**17,669**	**100.0**	**73,913**	**100.0**	**113,325**	**100.0**

Note:

^&^not included de jure resident

All the covariates are tested with Pearson chi-squared test for SC, ST and non-SC/ST and found to be statistically significant at p< 0.001

In terms of environmental factors, the use of solid fuel for cooking is higher among the households of SC (83%) and ST (93%) children compared to those of non-SC/ST (71%) children. The use of unimproved sanitation facilities and unsafe sources of drinking water is substantially higher among ST households compared to non-SC/ST households. As far as program indicators are concerned, the proportion of institutional delivery is lower, and the number of “never visit” responses for ANC during pregnancy is higher among SCs and STs compared to those of the non-SC/ST population. It is worth to mention that the ANC does not reach the aim of at least three visits for a large part of the population in the high focus states of India.

### Association between U5MR and different demographic, socioeconomic and program indicators

Table 3 presents the odds ratios for the predictors of under-five deaths. Model 1 is a basic model that includes the only caste as a covariate. In this model, compared to non-SC/ST children, SC (OR: 1.31, p<0.01, SE: 0.05) and ST (OR: 1.09, p<0.1, SE: 0.05) children have a significantly higher likelihood of dying. In model 2, along with the basic model, a set of socioeconomic factors are included in the model. Their addition results in a significant decrease from basic model in the odds of under-five deaths for children belonging to SC (OR: 1.15, p<0.01, SE: 0.05) and ST (OR: 0.89, p<0.05, SE: 0.04) compared to children belonging to non-SC/ST, demonstrating that socioeconomic differentials are at least partly responsible for the caste mortality differential. In model 3, a set of demographic factors and program indicators are included in the basic model and considered it as a proximate model to test if these factors can explain the caste mortality. The inclusion of these factors reduces the difference between non-SC/ST and SC but no significant difference between non-SC/ST and ST. Model 4 is the full model where all the basic, socioeconomic and proximate factors are included, and results revealed a significant decrease in the odds of child death for SC and ST compared to non-SC/ST children. Thus, results show that both socioeconomic and proximate factors are essential to explain the caste differentials in under-five death risk.

**Table 3:** Logit estimates of under-five death risk by different characteristics of high focus states in India, 2015–16.

Variables	Under-five mortality			
	Unadjusted OR (Model 1-basic)	Adjusted OR (Model 2-socioeconomic)	Adjusted OR (Model 3-proximate)	Adjusted OR (Model 4-full model)
**Caste**				
Non-SC/ST(Ref)				
SC	1.31***(0.05)	1.15***(0.05)	1.23***(0.05)	1.14***(0.05)
ST	1.09*(0.05)	0.89**(0.04)	1.05(0.05)	0.92*(0.04)
**Sex of the child**				
Female (Ref)				
Male			1.01(0.03)	1.01(0.03)
**Mother’s age at first birth (yr)**				
<20(Ref)				
20–24			0.95(0.03)	0.97(0.03)
≥ 25			1.06(0.06)	1.13**(0.06)
**Birth order**				
1 (Ref)				
2–3			0.80***(0.03)	0.76***(0.03)
4+			1.39***(0.06)	1.19***(0.06)
**Mother’s level of education**				
No education (Ref)				
Primary		0.89***(0.04)		0.97(0.05)
Secondary		0.72***(0.03)		0.81***(0.04)
Higher		0.62***(0.05)		0.69***(0.06)
**Type of residence**				
Urban(Ref)				
Rural		1.00(0.05)		1.00(0.05)
**Wealth quintile (household)**				
Poorest (Ref)				
Poorer		0.91**(0.04)		0.96(0.04)
Middle		0.80***(0.04)		0.87***(0.05)
Richer		0.72***(0.05)		0.79***(0.05)
Richest		0.53***(0.05)		0.59***(0.05)
**Type of fuel used for cooking**				
Clean fuel & food not cooked (Ref)				
Solid fuel		0.92(0.05)		0.91(0.05)
**Type of toilet**				
Improved toilet (Ref)				
Not improved toilet		1.13***(0.05)		1.12***(0.05)
**Source of drinking water**				
Improved water (Ref)				
Not improved water		0.93(0.05)		0.93(0.05)
**Place of birth**				
Home delivery(Ref)				
Institutional delivery			0.87***(0.03)	0.92**(0.03)
**ANC visits during pregnancy (last birth)**				
None (Ref)				
1 visit			0.85**(0.06)	0.87**(0.06)
2–3 visits			0.80***(0.03)	0.85***(0.04)
4+ visits			0.64***(0.03)	0.74***(0.03)
**Constant**	0.03***(0.00)	0.05***(0.00)	0.05***(0.00)	0.06***(0.01)
**Log Likelihood**	-17479	-17293	-17275	-17190
**Observation**	113,325	113,325	113,325	113,325

Notes: OR-Odds ratio

*** p<0.01

** p<0.05

* p<0.1; The entries in parenthesis refer to standard errors

Ref- indicate reference category

The persistence of the significant effect of SCs on under-five deaths is seen in the four models presented in Table 3; however, effect of ST category has diminished after the introduction of socioeconomic and proximate factors in the models. This supports our hypothesis that caste has an independent effect on under-five deaths and differential can be attributed by the socioeconomic, and proximate factors. It suggests that socioeconomic condition and program indicators may play a more critical role in the difference between deprived and other caste groups in the high focus states of India.

Further, the effect of selected factors within caste group is present in Table 4. The results revealed that for all the caste group, children are less likely to die when their mothers have completed secondary or higher education than children born to mothers who have no education. In case of household wealth, as the level of wealth of the household increases, the odds of under-five deaths is significantly decreases for SCs and non-SCs/STs, however, no significant effect is observed in case of children belonging to ST. The proximate factors like birth order and ANC visit are significantly associated with under-five death reduction for all the caste group. The sex of the child and, age of mothers at the birth of their first child were found to be significantly associated with under-five death risk in case of ST children. Place of birth was found to be a significant predictor in reducing under-five death risk among ST children. The likelihood of lower under-five death increased when mothers were able to opt for institutional delivery. Among the environmental factors, ST children belonging to household using solid fuel for cooking have a higher likelihood of under-five death.

**Table 4:** Logit estimates of under-five death risk for caste group by different characteristics of high focus states in India, 2015–16.

Variables	Odds ratio		
	SC	ST	Non-SC/ST
**Sex of the child**			
Female (Ref)			
Male	1.11(0.07)	1.22**(0.10)	0.93*(0.04)
**Mother’s age at first birth (yr)**			
<20(Ref)			
20–24	1.01(0.07)	0.98(0.09)	0.95(0.04)
≥ 25	1.09(0.13)	1.27*(0.16)	1.11(0.07)
**Birth order**			
1 (Ref)			
2–3	0.73***(0.06)	0.63***(0.06)	0.81***(0.04)
4+	1.01(0.10)	0.81*(0.09)	1.41***(0.08)
**Mother’s level of education**			
No education (Ref)			
Primary	0.95(0.09)	1.09(0.12)	0.94(0.06)
Secondary	0.85*(0.08)	0.84(0.09)	0.79***(0.04)
Higher	0.89(0.17)	0.50*(0.19)	0.67***(0.07)
**Type of residence**			
Urban(Ref)			
Rural	1.18(0.14)	0.88(0.16)	0.97(0.06)
**Wealth quintile (household)**			
Poorest (Ref)			
Poorer	0.96(0.08)	0.95(0.10)	0.97(0.06)
Middle	0.68***(0.08)	0.88(0.15)	0.95(0.06)
Richer	0.61***(0.10)	1.09(0.25)	0.85**(0.07)
Richest	0.40***(0.09)	0.67(0.28)	0.66***(0.07)
**Type of fuel used for cooking**			
Clean fuel & food not cooked (Ref)			
Solid fuel	0.87(0.11)	2.49***(0.75)	0.87**(0.06)
**Type of toilet**			
Improved toilet (Ref)			
Not improved toilet	0.99(0.10)	0.96(0.12)	1.20***(0.06)
**Source of drinking water**			
Improved water (Ref)			
Not improved water	0.77(0.10)	1.01(0.10)	0.94(0.07)
**Place of birth**			
Home delivery(Ref)			
Institutional delivery	0.97(0.07)	0.67***(0.06)	0.99(0.05)
**ANC visits during pregnancy (last birth)**			
None (Ref)			
1 visit	0.89(0.12)	0.98(0.18)	0.85**(0.07)
2–3 visits	0.88(0.07)	0.90(0.09)	0.83***(0.04)
4+ visits	0.80**(0.08)	0.78**(0.09)	0.71***(0.04)
**Constant**	0.07***(0.01)	0.03***(0.01)	0.06***(0.01)
**Log Likelihood**	-3823	-2701	-10619
**Observation**	21,743	17,669	73,913

Notes:

*** p<0.01

** p<0.05

* p<0.1

The entries in parenthesis refer to standard errors; Ref- indicate reference category

### Results of the decomposition analysis

The results of the detailed Fairlie decomposition is present in Table 5. Summary results of the decomposition analysis indicate that after controlling other factors, the likelihood of under-five death is higher among SCs/STs than among the non-SC/ST population. Results further indicate that the factors included in the analysis can explain 76% of the under-five mortality gap between SCs/STs and the non-SC/ST population. Of the explained gap, 83% can be related to differences in the distribution of women’s educational attainment and household wealth for under-five death risk. The unexplained gap (24%) for under-five death risk might be related to other structural factors not covered by the dataset in the analysis.

**Table 5:** Results of Fairlie decomposition of average gap in under-five death risk between caste groups in the high focus states of India, 2015–16.

Covariates	U5MR	
	Contribution coefficient	Contribution coefficient in percentages
**Mother’s level of education**	0.935**	18%
**Mother’s age at first birth**	-0.143	-3%
**Sex of the child**	-0.205**	-4%
**Birth order**	-0.087	-2%
**Wealth of the household**	3.300***	65%
**Type of fuel used for cooking**	0.448	9%
**Type of toilet**	-0.142	-3%
**Source of drinking water**	-0.292**	-6%
**Type of residence**	0.188	4%
**Place of birth**	0.525**	10%
**ANC visits**	0.556***	11%
**Summary of Fairlie decomposition(express in per 1000)**		
**Mean prediction of SC/ST**	40.2	
**Mean prediction of non-SC/ST**	33.5	
**Row differentials**	6.7	
**Total explained**	5.1	
**% Explained gap in U5M between SC/ST and non-SC/ST**	**76%**	
**% Unexplained gap in U5M between SC/ST and non-SC/ST**	**24%**	
**Number of observations**	**113,325**	

Note:

*** p< 0.01

** p< 0.05

* p< 0.10

In Table 5, the positive contribution of a covariate indicates that this particular covariate contributed to widening the under-five death risk gap between SCs/STs and the non-SC/ST populations, while the negative contribution of a covariate indicates that it was helping to reduce the gap. Household wealth is the most significant contributor (65%) to the gap in under-five death risk between SCs and STs, followed by the mother’s level of educational attainment (18%). Program indicators such as place of birth (10%) and the number of ANC visits (11%) also contribute to widening the under-five death risk gap between SCs/STs and the non-SC/ST population significantly. Demographic variables such as the sex of the child, the mother’s age at the birth of her first child and birth order contributed to reducing the gap. While the source of drinking water narrowed the gap (6%), the type of toilet used in the household diminished the caste gap in under-five death risk, although not significantly. The type of fuel used for cooking and the type of residence widened the caste gap in under-five death risk, although its contributions are not statistically significant.

## Discussion and conclusion

Any country’s general medical and public health conditions, and consequently its level of socioeconomic development, can be measured based on the health of its children. Although U5MR has been continuously declining in India in recent decades, it is still substantially higher among certain social groups and regions in the country, particularly before the implementation of the NRHM. This study documented disparities inU5MR by caste groups in the high mortality regions of India in recent years, using nationally representative data. The novelty of the study lies therein that, to our knowledge, this is the first study in India that provides district-level estimates of U5MR and systematically investigates the factors explaining under-five death risk by caste groups using the most recent DHS data. Also, it is the first study to document the association between caste and under-five death risk in the post-NRHM period in India.

Our study highlights a few important findings. First, the disparity in U5MR was profound by caste groups during the pre-NRHM period. This disparity has been reduced drastically in recent years. This success may be attributed to the NRHM under which special provision has been made for maternal and child health care services for women and children belonging to deprived castes. For instance, under the NHM, a conditional cash transfer scheme was introduced for institutional deliveries. In the high performing states of India, this cash was given only to women from deprived caste groups, whereas in low performing states, this cash was available to all poor as well as deprived castes women [39–41]. Despite the reduction in U5MR between caste groups over time, our study reveals that the caste gap in U5MR is persistent even in the NRHM/NHM period. Children belonging to deprived castes have a higher likelihood of dying than those belonging to non-deprived castes. Our analysis shows that this association remains significant for children from SCs even after controlling for other background characteristics. Thus, our study reconfirms that children belonging to deprived castes are still in a disadvantaged position in terms of mortality outcomes. These findings are consistent with those of previous national and sub-national studies [16,17,21,42]. Our results also indicate that the U5MR of the SC/ST population at district level is higher than that of the non-SC/ST population. We observed a geographical clustering of U5MR in the studied area.

Secondly, regression results revealed that educational attainment of mothers is statistically significantly associated with under-five death risk. As expected, under-five death risk shows significant declines with increases in the educational level of the mothers. Similar results observed for household wealth. As the level of wealth of the household increases, the odds of under-five deaths significantly decrease. Numerous previous studies document the negative and strong role of education on mortality through various mechanisms such as increased knowledge, better accessibility, use of modern health care facilities, higher mobility so on [43–47]. Educated mothers are more likely to develop good health-seeking behavior for themselves and their children, especially in case of utilization of health services, feeding and child care practices, concern about the hygienic condition which in turn will result in better health outcomes for both mothers and their children. Mother’s education could modify her role in the family and urge her to take critical measure to good child health outcomes and effective utilization of health services, which will, affects her economic development and her family as well. Maternal education also indicates early life and childhood environment of the mother affecting her adult health and economic condition. Some previous studies established that maternal education is the single most important determinant of child survival at all levels, and its association on child survival are stronger than those of household wealth [48–50]. A study based on 42 developing countries, argued that higher education levels were associated with disproportionately greater returns to child health [51]. Our study in India’s high mortality areas supports previous findings that maternal education and household wealth index are the crucial determinants of under-five mortality.

Our regression analysis also revealed that the household environmental factors, the type of toilet used is significantly associated with under-five death risk. The likelihood of under-five death is higher among children from households with non-improved toilet facilities compared to children of households with improved toilet facilities. Similarly, among the program indicators, place of birth, and the number of ANC visits during pregnancy were found to be significant indicators in reducing under-five deaths in the highly focused states of India.

The decomposition analysis allows us to gain insight into the relative contribution of various factors in the caste-based gaps in the U5MR. It demonstrates that the current gap in under-five death risk by caste groups is primarily due to their disadvantages in terms of the economic conditions and educational status of their mothers. This is consistent with the findings of previous research [23,52–55]. This indicates that there is a greater economic divide between well-off and deprived caste groups, which explains the majority of the gap in under-five death risk between caste groups. Since more than 50% of SC/ST households are from the most impoverished backgrounds, it is not surprising that household economic status turned out to be the largest contributing factor in widening the caste gap in under-five deaths. It is argued that poor SC/ST households do not have enough resources for child and maternal health care expenses. In contrast, the non-SC/ST population is economically better off and more educated. They may have a more advanced view, more knowledge about child care and preventive care (greatly associated with the modern healthcare system), as well as higher confidence in dealing with health care providers and a greater ability and readiness to travel outside the community for their health needs [52], all of which may help to reduce poor child health outcomes. The lower level of education among SCs and STs is accompanied by low awareness of health services. This includes less knowledge of the benefits of preventive child health care, use of traditional health care, poor communication with the husband and other family members on health-related issues and poor decision-making power within the family, low self-confidence, poor survival abilities, and poor negotiating skills with health care providers [56].

The type of cooking fuel used by households also positively contributed to widening the gap, which is an indication of less access to clean fuel by the disadvantaged caste groups. It is interesting to note that the two program-related variables together contributed 21% to the gap in the under-five death risk between the groups. Since these program indicators are highly related with neonatal mortality, it is imperative to see (S2 Table) that institutional delivery and higher frequency of ANC visit substantially reduce the neonatal mortality rate in the study area. This is an interesting finding from a policy perspective as it shows that there is still scope to uplift access to health facilities for women and children belonging to deprived caste groups in the study area.

Despite the U5MR of our study area experiencing a faster decline in the most recent years compared to the stagnation in mortality reduction that was observed in the early 2000s [57], this reduction is still not enough to achieve the SDG goals on preventable neonatal and under-five deaths [3]. Previous findings indicate that nearly all districts of Uttar Pradesh, Bihar, Madhya Pradesh, and Chhattisgarh will fail to achieve the SDG3 goal on neonatal mortality rate [3]. Further, in Uttar Pradesh, not a single district is expected to meet the target for U5MR as set out in SDG3 [3]. Since our findings demonstrate that economic status and level of maternal education is relatively more effective in U5MR reduction, there is a greater need to raise the economic status and levels of educational attainment, particularly secondary education among girls in the high focus states of India. The programs uplifting the economic status of disadvantaged groups are essential for a faster and sustainable reduction of U5MR in the high focus states. To improved universal coverage and access to maternal and child health care services, emphasis should be placed on creating awareness of the district level intervention program through community-based awareness programs, as well as on educating parents about the possible high-risk factors and preventive measures associated with child health [3]. Another possible initiative might be to involve the parents of SC and ST children in health-related intervention programs at the village or community level and educate them about preventive care for their infants at home. Creating awareness around preventive health care, maternal care, nutrition, awareness about infectious diseases, the benefits of hygiene and sanitation, and subsidized maternal health care services among SC and ST populations, should be increased through outreach programs.

## Supporting information

S1 Table Estimated districtwise under-five mortality rate for ten-years periods preceding the survey for SC, ST, Non-SC/ST and total in high focus states of India, 2015–16. (PDF)

S2 Table Neonatal mortality rate (per 1000 live births) by place of birth and ANC visit for SC, ST and non-SC/ST population in high focus states of India, 2015–16. (DOCX)

S1 Appendix Description of Fairlie method. (DOCX)
