# Supplementary material for: The persistent influence of caste on under-five mortality: Factors that explain the caste-based gap in high focus Indian states
Source: PLoS One. 2019 Aug 20;14(8):e0211086. doi: 10.1371/journal.pone.0211086 (PMC6701792; doi:10.1371/journal.pone.0211086)
Supplement: S1 Table — (PDF) [file pone.0211086.s001.pdf]

S1 Table. Estimated Districtwise Under-five mortality rate for ten-years periods preceding the survey for SC,ST, Non-SC/ST and Total in high focus states of India, 2015-16.

State	District	SC				ST				Non-SC/ST				Total			
		U5MR	P value	95% CI		U5MR	P value	95% CI		U5MR	P value	95% CI		U5MR	P value	95% CI	
				Lower	Upper			Lower	Upper			Lower	Upper			Lower	Upper
Uttarakhand	Uttarkashi	46.9	0.003	16.4	77.3	NA	-	-	-	74.7	0.000	56.2	93.1	67.2	0.000	49.3	85.2
Uttarakhand	Chamoli	38.5	0.032	3.3	73.7	NA	-	-	-	43.0	0.000	27.3	58.8	41.5	0.000	26.5	56.6
Uttarakhand	Rudraprayag	37.9	0.004	12.4	63.3	NA	-	-	-	27.0	0.000	13.9	40.2	29.5	0.000	15.6	43.4
Uttarakhand	Tehri garhwal	35.5	0.008	9.1	61.9	NA	-	-	-	33.1	0.000	18.9	47.3	34.5	0.000	21.6	47.4
Uttarakhand	Dehradun	19.5	0.004	6.2	32.7	56.2	0.033	4.7	107.8	32.5	0.000	20.0	45.0	31.1	0.000	23.4	38.9
Uttarakhand	Garhwal	17.3	0.212	-9.9	44.5	NA	-	-	-	29.2	0.000	14.6	43.8	26.8	0.000	14.0	39.6
Uttarakhand	Pithoragarh	44.8	0.004	14.0	75.6	40.6	0.254	-29.1	110.2	43.0	0.000	24.0	61.9	43.4	0.000	27.4	59.3
Uttarakhand	Bageshwar	63.8	0.000	32.5	95.0	NA	-	-	-	54.8	0.000	33.1	76.5	56.8	0.000	40.2	73.5
Uttarakhand	Almora	38.8	0.012	8.5	69.2	NA	-	-	-	48.6	0.000	23.9	73.2	46.2	0.000	28.5	63.9
Uttarakhand	Champawat	59.6	0.000	29.4	89.7	111.4	0.117	-28.1	250.8	55.1	0.000	35.3	74.8	57.3	0.000	41.8	72.8
Uttarakhand	Nainital	61.8	0.000	38.6	85.1	33.9	0.363	-39.2	106.9	46.8	0.000	30.2	63.3	50.7	0.000	39.3	62.1
Uttarakhand	Udham singh nagar	55.7	0.000	33.7	77.7	33.1	0.009	8.3	58.0	48.2	0.000	34.2	62.2	48.2	0.000	37.2	59.3
Uttarakhand	Hardwar	92.0	0.000	65.7	118.4	46.4	0.375	-56.1	148.8	67.2	0.000	54.0	80.4	73.3	0.000	62.5	84.0
Rajasthan	Ganganagar	63.1	0.000	37.0	89.3	45.6	0.504	-88.2	179.3	33.6	0.000	17.7	49.5	47.4	0.000	33.4	61.4
Rajasthan	Hanumangarh	59.4	0.000	29.5	89.3	70.2	0.143	-23.8	164.1	36.0	0.000	18.9	53.1	43.7	0.000	28.5	58.9
Rajasthan	Bikaner	58.1	0.000	36.2	80.0	NA	-	-	-	46.7	0.000	36.3	57.1	48.8	0.000	40.8	56.8
Rajasthan	Churu	38.8	0.000	18.4	59.2	35.8	0.336	-37.1	108.8	37.8	0.000	24.7	50.8	38.0	0.000	27.4	48.6
Rajasthan	Jhunjhunun	51.9	0.003	18.2	85.6	98.1	0.157	-37.6	233.7	37.5	0.000	23.1	52.0	41.1	0.000	24.8	57.4
Rajasthan	Alwar	43.7	0.002	16.4	71.1	52.2	0.029	5.5	98.8	43.6	0.000	29.4	57.8	44.5	0.000	31.1	58.0
Rajasthan	Bharatpur	44.5	0.002	16.9	72.2	NA	-	-	-	50.0	0.000	35.1	64.8	47.0	0.000	34.9	59.1
Rajasthan	Dhaulpur	79.9	0.000	51.1	108.7	94.0	0.012	20.5	167.5	53.6	0.000	36.1	71.0	64.2	0.000	48.1	80.3
Rajasthan	Karauli	100.6	0.000	66.8	134.3	126.6	0.000	81.2	171.9	77.6	0.000	56.9	98.4	92.9	0.000	70.6	115.3
Rajasthan	Sawai madhopur	74.2	0.000	42.0	106.4	88.9	0.000	55.8	122.1	42.8	0.000	25.6	59.9	58.2	0.000	45.4	71.0
Rajasthan	Dausa	65.4	0.000	30.9	99.9	39.1	0.011	9.1	69.1	72.6	0.000	46.1	99.2	63.3	0.000	48.7	77.8
Rajasthan	Jaipur	89.2	0.000	62.0	116.4	59.4	0.001	24.8	94.1	50.5	0.000	39.7	61.2	60.5	0.000	48.4	72.6
Rajasthan	Sikar	61.2	0.001	26.3	96.0	25.7	0.343	-27.4	78.7	36.8	0.000	21.7	51.9	41.3	0.000	27.1	55.4
Rajasthan	Nagaur	55.1	0.000	28.6	81.6	40.8	0.345	-43.9	125.4	26.9	0.000	13.0	40.8	34.8	0.000	21.4	48.2
Rajasthan	Jodhpur	87.1	0.000	55.4	118.7	36.4	0.022	5.4	67.5	47.5	0.000	35.6	59.5	54.6	0.000	44.2	64.9
Rajasthan	Jaisalmer	52.6	0.003	17.6	87.6	41.8	0.023	5.9	77.7	58.2	0.000	41.5	74.9	55.4	0.000	38.6	72.2
Rajasthan	Barmer	76.4	0.000	51.4	101.4	82.9	0.045	1.8	163.9	60.4	0.000	45.3	75.4	64.9	0.000	49.7	80.1
Rajasthan	Jalor	82.5	0.000	44.3	120.8	58.4	0.004	18.6	98.1	59.8	0.000	41.9	77.7	63.4	0.000	48.6	78.2
Rajasthan	Sirohi	101.3	0.000	53.7	149.0	70.4	0.000	40.0	100.8	57.6	0.000	37.2	78.0	70.3	0.000	56.3	84.3
Rajasthan	Pali	76.9	0.000	38.0	115.7	62.2	0.010	15.0	109.4	59.7	0.000	43.7	75.7	63.8	0.000	44.8	82.8
Rajasthan	Ajmer	59.8	0.000	37.2	82.4	39.2	0.131	-11.7	90.0	31.2	0.000	21.0	41.3	38.2	0.000	27.3	49.1
Rajasthan	Tonk	84.2	0.000	45.1	123.4	13.7	0.145	-4.7	32.1	46.7	0.000	31.5	62.0	50.4	0.000	33.1	67.8
Rajasthan	Bundi	82.7	0.000	41.3	124.1	60.6	0.000	26.7	94.6	51.4	0.000	32.9	69.9	61.0	0.000	44.0	78.1
Rajasthan	Bhilwara	25.2	0.071	-2.1	52.6	103.5	0.001	43.4	163.7	46.6	0.000	30.4	62.7	48.5	0.000	32.1	64.8
Rajasthan	Rajsamand	62.8	0.007	16.8	108.7	61.0	0.000	27.4	94.5	65.2	0.000	47.5	82.8	64.1	0.000	48.5	79.7
Rajasthan	Dungarpur	16.1	0.322	-15.8	48.1	48.9	0.000	33.3	64.6	28.0	0.015	5.4	50.6	42.6	0.000	30.1	55.2
Rajasthan	Banswara	97.8	0.048	0.8	194.8	39.7	0.000	26.5	52.9	44.2	0.008	11.4	76.9	41.8	0.000	30.2	53.4
Rajasthan	Chittaurgarh	55.1	0.002	20.0	90.2	53.1	0.008	13.6	92.6	55.3	0.000	30.1	80.4	54.3	0.000	38.5	70.2
Rajasthan	Kota	43.8	0.000	23.7	63.9	55.7	0.001	24.2	87.2	38.8	0.000	27.1	50.6	42.0	0.000	32.5	51.5
Rajasthan	Baran	43.8	0.002	15.9	71.8	72.8	0.002	26.1	119.6	45.8	0.000	26.4	65.2	49.8	0.000	33.2	66.5
Rajasthan	Jhalawar	59.0	0.015	11.5	106.4	46.1	0.001	18.3	73.9	74.8	0.000	48.7	100.9	64.8	0.000	47.5	82.1
Rajasthan	Udaipur	25.7	0.336	-26.6	77.9	74.8	0.000	49.5	100.1	38.1	0.002	14.5	61.7	61.3	0.000	46.1	76.5
Rajasthan	Pratapgarh	94.8	0.010	22.7	166.8	66.9	0.000	49.6	84.2	51.4	0.000	27.9	74.9	64.7	0.000	51.4	78.0
Uttar Pradesh	Saharanpur	75.8	0.000	56.0	95.6	NA	-	-	-	76.1	0.000	62.4	89.8	76.0	0.000	62.7	89.3
Uttar Pradesh	Muzaffarnagar	55.4	0.010	13.2	97.6	NA	-	-	-	66.1	0.000	54.3	77.8	64.4	0.000	50.2	78.5
Uttar Pradesh	Bijnor	88.8	0.000	53.3	124.3	NA	-	-	-	53.2	0.000	38.2	68.1	60.5	0.000	44.3	76.7
Uttar Pradesh	Moradabad	104.4	0.000	73.5	135.2	NA	-	-	-	94.8	0.000	82.5	107.0	96.4	0.000	83.1	109.7
Uttar Pradesh	Rampur	62.8	0.001	24.3	101.3	NA	-	-	-	71.4	0.000	56.8	86.0	70.0	0.000	57.8	82.1
Uttar Pradesh	Jyotiba phule nagar	91.3	0.000	51.0	131.6	NA	-	-	-	82.1	0.000	63.8	100.4	82.9	0.000	67.7	98.0
Uttar Pradesh	Meerut	103.0	0.000	73.1	132.9	NA	-	-	-	71.0	0.000	55.7	86.3	78.1	0.000	66.8	89.4
Uttar Pradesh	Baghpat	69.3	0.004	22.1	116.5	NA	-	-	-	50.5	0.000	37.9	63.1	52.4	0.000	40.0	64.8
Uttar Pradesh	Ghaziabad	76.4	0.000	42.2	110.6	NA	-	-	-	71.6	0.000	53.4	89.9	72.1	0.000	58.5	85.7
Uttar Pradesh	Gautam buddha nagar	73.9	0.000	45.7	102.1	NA	-	-	-	71.1	0.000	53.5	88.7	71.3	0.000	58.2	84.5
Uttar Pradesh	Bulandshahr	59.4	0.000	29.3	89.5	NA	-	-	-	78.2	0.000	59.0	97.4	73.0	0.000	53.8	92.2
Uttar Pradesh	Aligarh	116.7	0.000	93.6	139.9	NA	-	-	-	88.2	0.000	76.4	100.1	95.8	0.000	83.4	108.3
Uttar Pradesh	Mahamaya nagar	94.5	0.000	51.3	137.6	NA	-	-	-	84.2	0.000	65.0	103.4	86.4	0.000	67.4	105.3
Uttar Pradesh	Mathura	139.3	0.000	99.2	179.3	NA	-	-	-	61.7	0.000	45.2	78.2	79.7	0.000	62.8	96.6
Uttar Pradesh	Agra	81.2	0.000	61.7	100.6	200.6	0.021	30.2	371.0	71.9	0.000	59.1	84.7	75.5	0.000	65.4	85.5
Uttar Pradesh	Firozabad	66.0	0.000	43.7	88.3	NA	-	-	-	93.2	0.000	78.2	108.2	87.1	0.000	74.9	99.2
Uttar Pradesh	Mainpuri	107.0	0.000	66.5	147.5	NA	-	-	-	92.0	0.000	70.5	113.5	95.6	0.000	78.8	112.3
Uttar Pradesh	Budaun	132.7	0.000	96.3	169.1	NA	-	-	-	112.8	0.000	93.5	132.2	117.3	0.000	101.6	133.0
Uttar Pradesh	Bareilly	89.2	0.000	45.7	132.6	NA	-	-	-	85.7	0.000	73.0	98.5	85.8	0.000	76.0	95.7
Uttar Pradesh	Pilibhit	97.2	0.000	46.3	148.1	NA	-	-	-	98.9	0.000	77.2	120.6	97.8	0.000	80.1	115.6
Uttar Pradesh	Shahjahanpur	103.0	0.000	63.7	142.3	NA	-	-	-	103.8	0.000	82.6	125.1	103.4	0.000	84.8	122.0
Uttar Pradesh	Kheri	133.5	0.000	97.2	169.7	92.9	0.042	3.2	182.6	95.6	0.000	75.6	115.7	107.5	0.000	89.9	125.2
Uttar Pradesh	Sitapur	100.8	0.000	78.0	123.6	NA	-	-	-	120.3	0.000	94.1	146.5	112.2	0.000	92.5	132.0
Uttar Pradesh	Hardoi	72.6	0.000	43.9	101.4	NA	-	-	-	99.3	0.000	73.1	125.5	89.1	0.000	72.1	106.0
Uttar Pradesh	Unnao	77.0	0.000	46.3	107.8	NA	-	-	-	80.1	0.000	55.7	104.5	78.5	0.000	64.2	92.9
Uttar Pradesh	Lucknow	63.8	0.000	38.7	89.0	NA	-	-	-	49.6	0.000	34.6	64.6	53.8	0.000	40.2	67.5
Uttar Pradesh	Rae bareli	81.1	0.000	53.8	108.4	NA	-	-	-	87.6	0.000	63.9	111.3	84.3	0.000	63.9	104.7
Uttar Pradesh	Farrukhabad	144.5	0.000	90.5	198.6	NA	-	-	-	91.5	0.000	73.4	109.7	99.8	0.000	83.2	116.3
Uttar Pradesh	Kannauj	105.2	0.000	62.4	148.0	333.3	0.128	-95.8	762.5	94.2	0.000	77.5	110.9	96.8	0.000	79.2	114.5
Uttar Pradesh	Etawah	75.8	0.000	45.9	105.6	NA	-	-	-	74.1	0.000	56.3	91.9	73.8	0.000	54.3	93.3
Uttar Pradesh	Auraiya	80.4	0.000	45.2	115.7	NA	-	-	-	48.9	0.000	30.2	67.7	59.0	0.000	43.3	74.6
Uttar Pradesh	Kanpur dehat	94.9	0.000	64.5	125.4	NA	-	-	-	52.1	0.000	35.2	69.1	69.2	0.000	55.0	83.3
Uttar Pradesh	Kanpur nagar	56.7	0.000	28.2	85.3	113.5	0.382	-140.8	367.8	57.2	0.000	36.1	78.2	57.3	0.000	43.0	71.6
Uttar Pradesh	Jalaun	102.7	0.000	58.2	147.1	NA	-	-	-	63.5	0.000	41.2	85.7	72.7	0.000	49.8	95.6
Uttar Pradesh	Jhansi	99.2	0.000	69.6	128.9	175.0	0.026	20.9	329.0	80.5	0.000	62.4	98.7	88.7	0.000	75.5	102.0
Uttar Pradesh	Lalitpur	90.4	0.000	57.4	123.5	95.8	0.055	-2.2	193.7	71.8	0.000	48.5	95.1	77.7	0.000	58.1	97.3
Uttar Pradesh	Hamirpur	80.0	0.000	45.1	115.0	NA	-	-	-	40.9	0.000	24.3	57.5	51.0	0.000	33.2	68.9
Uttar Pradesh	Mahoba	70.1	0.002	25.2	115.1	NA	-	-	-	63.5	0.000	44.4	82.6	65.1	0.000	46.6	83.6
Uttar Pradesh	Banda	70.9	0.000	33.3	108.5	NA	-	-	-	69.6	0.000	47.2	91.9	69.4	0.000	51.1	87.8
Uttar Pradesh	Chitrakoot	99.3	0.000	71.6	127.0	76.9	0.092	-12.5	166.3	82.6	0.000	59.1	106.0	87.6	0.000	70.5	104.7
Uttar Pradesh	Fatehpur	91.2	0.000	57.1	125.3	NA	-	-	-	64.4	0.000	38.1	90.7	73.4	0.000	55.1	91.7
Uttar Pradesh	Pratapgarh	95.1	0.000	49.4	140.8	100.9	0.147	-35.3	237.1	68.5	0.000	45.3	91.6	74.3	0.000	57.3	91.3
Uttar Pradesh	Kaushambi	150.9	0.000	122.3	179.5	NA	-	-	-	94.4	0.000	69.4	119.4	121.2	0.000	100.6	141.8
Uttar Pradesh	Allahabad	75.2	0.000	41.9	108.5	118.2	0.261	-87.7	324.2	70.7	0.000	51.0	90.3	72.1	0.000	57.1	87.2
Uttar Pradesh	Bara banki	64.2	0.000	35.9	92.5	NA	-	-	-	62.1	0.000	41.8	82.4	61.0	0.000	44.2	77.7
Uttar Pradesh	Faizabad	85.5	0.000	56.1	114.9	NA	-	-	-	68.4	0.000	52.5	84.3	73.6	0.000	54.9	92.2
Uttar Pradesh	Ambedkar nagar	103.2	0.000	67.9	138.4	179.8	0.138	-57.6	417.3	66.8	0.000	46.9	86.7	79.2	0.000	62.3	96.0
Uttar Pradesh	Sultanpur	73.4	0.000	40.3	106.5	129.8	0.187	-63.1	322.6	87.4	0.000	67.6	107.3	84.2	0.000	66.3	102.2
Uttar Pradesh	Bahraich	75.6	0.000	47.4	103.9	44.8	0.051	-0.2	89.8	88.3	0.000	70.3	106.4	82.3	0.000	67.8	96.8
Uttar Pradesh	Shrawasti	154.6	0.000	114.9	194.4	143.6	0.052	-1.0	288.2	108.1	0.000	89.3	127.0	119.1	0.000	102.7	135.4
Uttar Pradesh	Balrampur	62.2	0.000	34.4	89.9	70.3	0.110	-15.8	156.3	70.3	0.000	53.3	87.4	68.7	0.000	53.5	83.9
Uttar Pradesh	Gonda	104.8	0.000	67.9	141.6	189.9	0.037	11.7	368.1	118.2	0.000	98.5	137.9	116.7	0.000	95.2	138.2
Uttar Pradesh	Siddharth nagar	75.4	0.000	40.4	110.3	157.1	0.050	-0.3	314.5	97.3	0.000	79.9	114.6	93.4	0.000	78.0	108.8
Uttar Pradesh	Basti	89.5	0.000	53.7	125.3	NA	-	-	-	78.6	0.000	59.1	98.0	80.6	0.000	65.8	95.3
Uttar Pradesh	Sant kabir nagar	92.2	0.000	63.9	120.5	NA	-	-	-	63.1	0.000	45.7	80.6	71.0	0.000	55.7	86.3
Uttar Pradesh	Mahrajganj	69.4	0.000	35.9	102.8	73.6	0.126	-20.7	167.8	68.2	0.000	53.3	83.0	68.6	0.000	55.2	81.9
Uttar Pradesh	Gorakhpur	61.2	0.000	32.9	89.5	NA	-	-	-	68.6	0.000	51.0	86.2	66.2	0.000	52.1	80.2
Uttar Pradesh	Kushinagar	68.0	0.000	35.9	100.0	122.0	0.107	-26.2	270.2	63.0	0.000	48.3	77.7	65.3	0.000	53.4	77.2
Uttar Pradesh	Deoria	110.5	0.000	76.1	144.9	58.9	0.126	-16.6	134.4	57.1	0.000	41.0	73.3	69.0	0.000	53.5	84.5
Uttar Pradesh	Azamgarh	76.3	0.000	51.3	101.3	NA	-	-	-	61.6	0.000	43.9	79.4	65.8	0.000	50.8	80.7
Uttar Pradesh	Mau	91.5	0.000	56.7	126.3	118.9	0.137	-37.7	275.5	63.0	0.000	45.6	80.4	70.9	0.000	54.9	86.9
Uttar Pradesh	Ballia	64.9	0.000	28.7	101.2	106.9	0.019	17.5	196.3	74.5	0.000	55.4	93.6	73.9	0.000	57.9	90.0
Uttar Pradesh	Jaunpur	118.2	0.000	81.1	155.3	55.3	0.430	-82.0	192.6	66.3	0.000	48.6	84.1	79.3	0.000	63.8	94.7

Uttar Pradesh	Ghazipur	95.6	0.000	64.8	126.3	NA	-	-	-	62.9	0.000	46.4	79.3	73.3	0.000	58.6	88.1
Uttar Pradesh	Chandauli	74.7	0.000	52.3	97.1	117.6	0.026	13.7	221.4	48.8	0.000	33.5	64.1	58.6	0.000	44.1	73.1
Uttar Pradesh	Varanasi	97.7	0.000	60.4	134.9	70.9	0.140	-23.4	165.2	56.3	0.000	44.5	68.2	64.3	0.000	50.0	78.6
Uttar Pradesh	Sant ravidas nagar (bhadohi)	104.2	0.000	72.5	135.9	77.0	0.316	-73.6	227.5	101.9	0.000	84.7	119.1	102.2	0.000	83.6	120.9
Uttar Pradesh	Mirzapur	124.7	0.000	91.6	157.9	28.8	0.100	-5.5	63.2	66.4	0.000	42.9	90.0	83.3	0.000	70.4	96.1
Uttar Pradesh	Sonbhadra	78.3	0.000	44.6	112.0	62.4	0.000	33.0	91.9	58.5	0.000	39.3	77.7	64.8	0.000	48.7	80.8
Uttar Pradesh	Etah	143.5	0.000	104.3	182.6	NA	-	-	-	81.0	0.000	62.7	99.4	93.7	0.000	79.3	108.1
Uttar Pradesh	Kanshiram nagar	130.3	0.000	88.8	171.8	NA	-	-	-	109.1	0.000	89.2	128.9	113.3	0.000	95.6	130.9
Bihar	Pashchim champaran	90.4	0.000	56.0	124.9	50.1	0.024	6.5	93.6	46.7	0.000	32.6	60.8	54.5	0.000	42.3	66.7
Bihar	Purba champaran	87.6	0.000	50.7	124.6	15.3	0.169	-6.5	37.2	76.2	0.000	57.5	94.9	73.5	0.000	56.9	90.1
Bihar	Sheohar	74.9	0.000	40.3	109.5	161.6	0.002	58.8	264.3	81.4	0.000	63.5	99.3	84.0	0.000	69.8	98.3
Bihar	Sitamarhi	105.2	0.000	72.3	138.2	72.5	0.072	-6.4	151.3	65.8	0.000	50.7	80.9	74.3	0.000	57.9	90.7
Bihar	Madhubani	56.0	0.000	32.3	79.6	NA	-	-	-	63.2	0.000	45.7	80.7	58.8	0.000	45.1	72.5
Bihar	Supaul	63.5	0.000	35.4	91.5	35.7	0.145	-12.4	83.7	51.2	0.000	38.2	64.1	53.6	0.000	42.8	64.5
Bihar	Araria	98.4	0.000	59.7	137.0	30.4	0.303	-27.5	88.2	77.5	0.000	59.7	95.3	80.7	0.000	64.0	97.4
Bihar	Kishanganj	53.6	0.017	9.7	97.5	42.7	0.019	6.9	78.5	51.2	0.000	38.3	64.1	50.8	0.000	37.6	64.0
Bihar	Purnia	98.0	0.000	58.2	137.8	61.5	0.144	-21.0	144.0	78.7	0.000	62.1	95.3	81.8	0.000	68.4	95.3
Bihar	Katihar	104.9	0.000	67.3	142.6	184.7	0.000	104.4	265.0	50.6	0.000	36.0	65.1	68.9	0.000	54.0	83.7
Bihar	Madhepura	76.0	0.000	48.6	103.5	85.4	0.021	13.2	157.7	60.6	0.000	46.4	74.9	65.4	0.000	54.0	76.9
Bihar	Saharsa	69.8	0.000	41.3	98.3	NA	-	-	-	66.5	0.000	52.6	80.3	67.0	0.000	54.2	79.9
Bihar	Darbhanga	61.5	0.000	34.9	88.1	50.1	0.010	12.1	88.1	29.2	0.000	16.1	42.2	39.1	0.000	29.2	49.0
Bihar	Muzaffarpur	52.8	0.000	26.0	79.6	NA	-	-	-	39.7	0.000	31.2	48.3	41.6	0.000	31.3	52.0
Bihar	Gopalganj	61.7	0.000	32.3	91.0	54.0	0.008	13.8	94.2	46.2	0.000	32.8	59.5	49.8	0.000	37.2	62.4
Bihar	Siwan	62.8	0.000	36.4	89.2	73.6	0.004	22.9	124.3	40.1	0.000	28.4	51.7	48.5	0.000	36.5	60.6
Bihar	Saran	56.6	0.001	23.4	89.8	NA	-	-	-	48.5	0.000	33.4	63.6	48.8	0.000	37.6	60.0
Bihar	Vaishali	56.6	0.000	32.3	80.9	108.6	0.018	18.7	198.5	56.5	0.000	41.3	71.8	58.5	0.000	44.1	72.9
Bihar	Samastipur	47.2	0.000	22.5	71.9	69.3	0.006	19.5	119.2	43.9	0.000	31.4	56.5	45.9	0.000	35.9	55.9
Bihar	Begusarai	60.4	0.000	30.1	90.7	92.6	0.283	-76.6	261.9	49.6	0.000	37.3	61.8	51.9	0.000	39.4	64.5
Bihar	Khagaria	45.3	0.000	20.9	69.8	200.0	0.356	-224.6	624.6	63.3	0.000	51.4	75.2	60.5	0.000	48.7	72.4
Bihar	Bhagalpur	42.7	0.003	14.4	71.1	15.1	0.300	-13.4	43.5	38.9	0.000	28.1	49.7	38.6	0.000	27.2	50.0
Bihar	Banka	98.9	0.000	56.6	141.2	59.6	0.031	5.4	113.7	45.8	0.000	35.1	56.6	54.8	0.000	44.0	65.6
Bihar	Munger	55.5	0.000	29.1	81.9	413.7	0.036	26.9	800.6	71.2	0.000	51.3	91.1	69.6	0.000	56.4	82.9
Bihar	Lakhisarai	89.9	0.000	52.5	127.4	51.3	0.106	-10.8	113.4	69.9	0.000	53.7	86.1	73.0	0.000	60.4	85.7
Bihar	Sheikhpura	74.0	0.000	50.2	97.8	NA	-	-	-	55.9	0.000	43.9	67.8	60.6	0.000	48.0	73.2
Bihar	Nalanda	57.8	0.000	34.5	81.2	59.9	0.359	-68.0	187.7	42.9	0.000	30.7	55.2	47.8	0.000	35.4	60.2
Bihar	Patna	73.6	0.000	50.5	96.6	NA	-	-	-	47.5	0.000	36.3	58.7	52.9	0.000	44.1	61.6
Bihar	Bhojpur	77.4	0.000	40.3	114.4	403.6	0.005	120.8	686.4	44.0	0.000	31.0	56.9	52.8	0.000	38.5	67.1
Bihar	Buxar	76.1	0.000	46.7	105.6	NA	-	-	-	60.0	0.000	46.0	74.0	62.7	0.000	47.0	78.4
Bihar	Kaimur (bhabua)	73.2	0.000	53.5	92.9	NA	-	-	-	77.0	0.000	57.7	96.3	75.1	0.000	62.8	87.4
Bihar	Rohtas	84.4	0.000	58.0	110.8	NA	-	-	-	57.9	0.000	40.8	75.0	64.6	0.000	50.7	78.5
Bihar	Aurangabad	51.4	0.000	24.4	78.3	NA	-	-	-	65.2	0.000	48.6	81.9	60.9	0.000	46.0	75.8
Bihar	Gaya	102.8	0.000	79.6	126.1	NA	-	-	-	59.5	0.000	44.0	75.0	74.5	0.000	59.9	89.2
Bihar	Nawada	63.7	0.000	37.5	89.9	NA	-	-	-	51.6	0.000	38.7	64.4	53.9	0.000	41.6	66.3
Bihar	Jamui	64.0	0.000	43.7	84.3	88.0	0.027	10.1	165.8	57.4	0.000	42.8	72.0	60.0	0.000	50.1	69.9
Bihar	Jehanabad	93.2	0.000	53.8	132.6	166.7	0.314	-158.1	491.4	51.9	0.000	35.9	67.8	63.6	0.000	44.7	82.5
Bihar	Arwal	56.3	0.000	32.4	80.2	NA	-	-	-	51.3	0.000	34.8	67.8	52.5	0.000	39.9	65.2
Assam	Kokrajhar	25.9	0.344	-27.7	79.5	45.2	0.001	19.3	71.2	61.5	0.000	38.8	84.1	54.8	0.000	39.0	70.6
Assam	Dhubri	69.5	0.122	-18.6	157.6	NA	-	-	-	44.8	0.000	32.3	57.2	45.3	0.000	31.8	58.8
Assam	Goalpara	16.3	0.368	-19.2	51.9	37.7	0.028	4.0	71.3	53.6	0.000	38.6	68.5	48.5	0.000	34.4	62.6
Assam	Barpeta	42.4	0.112	-10.0	94.9	NA	-	-	-	44.2	0.000	33.1	55.3	43.8	0.000	31.5	56.0
Assam	Morigaon	21.9	0.174	-9.6	53.4	90.4	0.000	44.5	136.3	78.7	0.000	58.9	98.5	75.2	0.000	58.6	91.9
Assam	Nagaon	34.5	0.083	-4.6	73.6	39.4	0.400	-52.3	131.0	74.7	0.000	55.4	94.0	70.0	0.000	52.9	87.0
Assam	Sonitpur	55.2	0.035	3.8	106.5	21.2	0.156	-8.1	50.5	53.5	0.000	37.2	69.8	49.3	0.000	34.7	64.0
Assam	Lakhimpur	92.0	0.012	20.6	163.3	36.9	0.006	10.5	63.3	51.7	0.000	30.1	73.3	50.1	0.000	33.1	67.1
Assam	Dhemaji	61.0	0.003	21.0	100.9	82.6	0.000	56.8	108.4	57.1	0.000	37.7	76.5	68.2	0.000	51.7	84.6
Assam	Tinsukia	25.5	0.139	-8.2	59.1	54.7	0.158	-21.2	130.5	89.9	0.000	68.0	111.8	82.7	0.000	66.1	99.3
Assam	Dibrugarh	NA	-	-	-	NA	-	-	-	33.0	0.000	19.1	46.8	28.9	0.000	16.0	41.9
Assam	Sivasagar	109.1	0.007	-	188.1	156.9	0.176	-70.5	384.2	73.4	0.000	55.2	91.5	77.3	0.000	63.6	91.1
Assam	Jorhat	58.0	0.037	3.6	112.5	31.2	0.037	1.9	60.5	55.1	0.000	34.3	76.0	52.2	0.000	33.5	70.8
Assam	Golaghat	23.9	0.359	-27.1	74.8	29.3	0.192	-14.7	73.3	57.2	0.000	38.3	76.0	52.1	0.000	35.1	69.2
Assam	Karbi anglong	69.4	0.024	9.1	129.7	48.2	0.000	32.2	64.1	113.2	0.000	75.9	150.6	70.7	0.000	55.0	86.3
Assam	Dima hasao	33.1	0.366	-38.6	104.8	77.8	0.000	57.6	97.9	80.5	0.000	41.1	119.9	76.7	0.000	56.8	96.6
Assam	Cachar	70.7	0.001	30.9	110.6	100.0	0.251	-70.8	270.8	78.6	0.000	58.1	99.0	77.3	0.000	59.0	95.7
Assam	Karimganj	144.6	0.000	76.1	213.1	13.8	0.345	-14.9	42.6	84.1	0.000	63.7	104.5	86.7	0.000	71.5	101.9
Assam	Hailakandi	57.5	0.001	24.5	90.4	NA	-	-	-	74.4	0.000	58.6	90.1	71.2	0.000	53.8	88.6
Assam	Bongaigaon	55.0	0.010	13.3	96.7	24.2	0.267	-18.5	66.9	31.2	0.000	17.2	45.3	35.1	0.000	20.4	49.8
Assam	Chirang	39.4	0.098	-7.2	86.1	61.5	0.000	29.6	93.4	47.2	0.000	25.5	68.9	50.6	0.000	34.4	66.8
Assam	Kamrup	15.0	0.303	-13.6	43.5	45.3	0.027	5.1	85.4	45.1	0.000	31.1	59.1	42.3	0.000	28.4	56.1
Assam	Kamrup metropolitan	64.4	0.001	25.8	102.9	28.0	0.185	-13.4	69.5	38.0	0.000	18.1	57.9	44.1	0.000	26.9	61.4
Assam	Nalbari	74.5	0.045	1.8	147.2	89.4	0.022	12.9	165.9	31.4	0.000	18.9	44.0	38.1	0.000	23.1	53.1
Assam	Baksa	30.8	0.072	-2.8	64.4	70.2	0.000	43.4	97.0	34.9	0.000	20.2	49.6	46.2	0.000	29.3	63.2
Assam	Darrang	109.3	0.050	-0.1	218.7	55.7	0.367	-65.2	176.5	61.9	0.000	45.3	78.5	63.7	0.000	47.2	80.2
Assam	Udalguri	9.9	0.209	-5.6	25.3	69.2	0.000	36.7	101.7	43.9	0.000	22.9	64.8	47.1	0.000	33.9	60.3
Jharkhand	Garhwa	83.6	0.001	34.4	132.8	47.5	0.003	16.5	78.5	67.6	0.000	44.3	90.9	67.5	0.000	50.5	84.6
Jharkhand	Chatra	111.4	0.000	81.9	141.0	78.0	0.001	30.2	125.7	53.3	0.000	35.1	71.5	75.9	0.000	61.8	90.0
Jharkhand	Kodarma	55.7	0.008	14.7	96.7	144.5	0.057	-4.2	293.1	34.9	0.000	22.4	47.5	40.4	0.000	26.9	53.8
Jharkhand	Giridih	98.6	0.001	39.3	157.9	71.9	0.001	29.4	114.4	51.5	0.000	37.4	65.7	59.0	0.000	46.8	71.2
Jharkhand	Deoghar	75.9	0.000	34.1	117.7	57.4	0.010	13.7	101.1	55.4	0.000	38.9	71.9	58.1	0.000	42.6	73.6
Jharkhand	Godda	85.7	0.001	33.1	138.2	71.8	0.000	39.6	104.0	68.1	0.000	47.1	89.1	71.2	0.000	52.0	90.5
Jharkhand	Sahibganj	53.6	0.016	10.2	97.1	94.9	0.000	66.8	123.1	48.1	0.000	32.0	64.2	61.7	0.000	48.2	75.1
Jharkhand	Pakur	44.0	0.074	-4.3	92.3	65.1	0.000	43.3	86.8	41.2	0.000	24.5	57.9	51.6	0.000	38.9	64.3
Jharkhand	Dhanbad	63.8	0.000	35.1	92.4	47.1	0.004	15.3	78.9	55.2	0.000	38.1	72.4	55.9	0.000	43.1	68.8
Jharkhand	Bokaro	55.1	0.000	35.4	74.9	41.6	0.001	16.0	67.1	43.3	0.000	29.5	57.0	46.4	0.000	35.2	57.5
Jharkhand	Lohardaga	10.2	0.306	-9.3	29.7	42.9	0.000	21.4	64.5	49.9	0.000	23.5	76.3	42.5	0.000	30.3	54.8
Jharkhand	Purbi singhbhum	18.9	0.069	-1.5	39.3	6.2	0.075	-0.6	12.9	17.6	0.000	8.5	26.7	13.2	0.000	7.6	18.9
Jharkhand	Palamu	97.6	0.000	56.9	138.3	47.9	0.017	8.6	87.1	48.4	0.000	30.4	66.5	61.6	0.000	44.1	79.1
Jharkhand	Latehar	59.7	0.001	25.8	93.7	36.5	0.000	22.0	50.9	28.4	0.000	12.8	44.0	38.8	0.000	27.3	50.2
Jharkhand	Hazaribagh	61.5	0.001	24.3	98.6	41.6	0.025	5.3	77.9	52.6	0.000	33.6	71.7	52.9	0.000	37.9	67.9
Jharkhand	Ramgarh	42.3	0.004	13.7	70.9	76.8	0.000	47.9	105.6	41.2	0.000	29.0	53.3	50.3	0.000	38.7	61.9
Jharkhand	Dumka	79.5	0.001	31.7	127.3	69.7	0.000	39.3	100.2	62.3	0.000	38.5	86.1	67.6	0.000	50.0	85.2
Jharkhand	Jamtara	36.8	0.029	3.8	69.7	96.0	0.000	58.0	134.0	45.6	0.000	27.3	63.9	56.1	0.000	39.5	72.7
Jharkhand	Ranchi	40.8	0.042	1.5	80.1	59.7	0.000	39.1	80.3	50.6	0.000	34.8	66.3	53.2	0.000	42.3	64.1
Jharkhand	Khunti	42.3	0.132	-12.7	97.3	39.2	0.000	21.4	57.0	51.4	0.000	24.7	78.0	42.4	0.000	29.1	55.7
Jharkhand	Gumla	61.9	0.008	15.8	108.0	45.0	0.000	29.0	61.1	40.4	0.000	17.7	63.0	45.1	0.000	30.4	59.9
Jharkhand	Simdega	120.8	0.001	51.1	190.4	110.0	0.000	86.9	133.2	60.2	0.001	25.4	95.0	98.9	0.000	82.4	115.3
Jharkhand	Pashchimi singhbhum	82.4	0.054	-1.3	166.1	110.8	0.000	89.6	132.1	82.3	0.000	38.7	126.0	105.3	0.000	87.6	123.0
Jharkhand	Saraikela kharsawan	41.3	0.096	-7.4	89.9	59.0	0.000	35.1	83.0	53.3	0.000	30.9	75.7	54.6	0.000	36.6	72.6
Odisha	Bargarh	48.5	0.018	8.4	88.7	21.0	0.154	-7.9	50.0	32.8	0.001	13.8	51.7	33.6	0.000	18.1	49.2
Odisha	Jharsuguda	30.0	0.008	7.9	52.2	68.3	0.000	42.6	94.0	52.0	0.000	35.9	68.1	53.5	0.000	41.9	65.2
Odisha	Sambalpur	91.6	0.000	50.9	132.3	55.2	0.006	15.6	94.9	28.7	0.021	4.3	53.1	56.2	0.000	35.4	77.0
Odisha	Debagarh	59.9	0.002	22.5	97.3	78.5	0.000	53.7	103.3	43.1	0.000	19.5	66.7	61.7	0.000	45.3	78.2
Odisha	Sundargarh	43.3	0.033	3.6	83.1	55.6	0.000	36.6	74.6	29.9	0.001	12.4	47.4	45.8	0.000	33.6	57.9
Odisha	Kendujhar	54.5	0.022	8.0	101.1	69.3	0.000	49.2	89.4	61.4	0.002	22.6	100.2	64.9	0.000	47.1	82.7
Odisha	Mayurbhanj	37.7	0.083	-4.9	80.2	78.9	0.000	57.4	100.3	23.9	0.054	-0.4	48.3	60.8	0.000	44.7	77.0
Odisha	Baleshwar	42.1	0.012	9.2	75.0	20.3	0.169	-8.6	49.2	33.8	0.001	13.8	53.9	34.1	0.000	19.5	48.7
Odisha	Bhadrak	50.5	0.006	14.5	86.5	168.4	0.001	72.1	264.6	28.3	0.000	12.9	43.7	43.7	0.000	27.0	60.4
Odisha	Kendrapara	21.2	0.062	-1.1	43.5	NA	-	-	-	58.2	0.000	40.4	76.0	46.0	0.000	32.9	59.1
Odisha	Jagatsinghapur	39.0	0.040	1.8	76.1	NA	-	-	-	45.3	0.000	24.2	66.4	43.3	0.000	24.8	61.9
Odisha	Cuttack	42.0	0.004	13.2	70.7	39.5	0.249	-27.7	106.8	24.7	0.002	9.2	40.3	29.2	0.000	13.2	45.2
Odisha	Jajapur	61.5	0.000	30.6	92.3	81.5	0.009	20.4	142.6	40.4	0.000	22.3	58.5	51.9	0.000	35.9	67.8
Odisha	Dhenkanal	54.7	0.000	25.2	84.3	86.3	0.002	31.6	141.1	38.9	0.001	16.8	60.9	51.1	0.000	36.3	66.0
Odisha	Anugul	60.2	0.001	23.1	97.3	68.5	0.000	33.7	103.2	58.2	0.000	37.3	79.0	61.1	0.000	41.0	81.1
Odisha	Nayagarh	134.6	0.000	75.3	194.0	92.8	0.003	31.3	154.3	52.9	0.000	31.7	74.1	72.2	0.000	53.6	90.9
Odisha	Khordha	42.1	0.027	4.7	79.5	38.6	0.064	-2.3	79.5	16.4	0.000	8.1	24.7	21.9	0.000	13.5	30.4
Odisha	Puri	33.0	0.022	4.7	61.3	NA	-	-	-	60.1	0.000	39.2	80.9	52.8	0.000	35.9	69.7

Odisha	Ganjam	63.3	0.000	30.7	95.9	142.1	0.012	31.0	253.1	24.3	0.000	11.6	37.0	41.4	0.000	26.7	56.0
Odisha	Gajapati	81.5	0.000	40.3	122.6	80.8	0.000	55.6	106.0	63.3	0.000	31.6	95.1	76.5	0.000	59.6	93.4
Odisha	Kandhamal	98.6	0.000	67.0	130.2	95.0	0.000	64.6	125.4	80.5	0.002	30.4	130.6	93.8	0.000	76.3	111.4
Odisha	Baudh	140.5	0.000	99.4	181.6	73.9	0.001	30.5	117.4	66.7	0.000	41.2	92.2	91.5	0.000	72.1	110.9
Odisha	Subarnapur	55.2	0.001	21.6	88.7	47.6	0.042	1.8	93.5	52.4	0.000	26.8	78.0	52.8	0.000	38.6	67.0
Odisha	Balangir	57.1	0.004	18.7	95.5	63.7	0.000	33.0	94.5	58.3	0.000	31.3	85.4	59.4	0.000	41.0	77.8
Odisha	Nuapada	65.7	0.006	18.4	112.9	93.0	0.000	58.5	127.5	35.7	0.000	16.6	54.8	60.2	0.000	44.2	76.1
Odisha	Kalahandi	82.7	0.000	45.7	119.7	73.9	0.000	33.9	114.0	75.0	0.000	45.7	104.3	76.8	0.000	54.2	99.4
Odisha	Rayagada	105.2	0.000	61.5	149.0	156.9	0.000	117.0	196.9	82.6	0.000	45.4	119.8	131.5	0.000	108.3	154.6
Odisha	Nabarangapur	62.8	0.000	29.9	95.8	85.5	0.000	64.9	106.2	108.1	0.000	62.3	153.9	86.7	0.000	68.5	104.8
Odisha	Koraput	74.9	0.001	32.1	117.7	63.0	0.000	38.8	87.2	64.8	0.000	31.8	97.8	65.5	0.000	47.3	83.6
Odisha	Malkangiri	66.9	0.000	32.2	101.7	110.7	0.000	86.3	135.1	61.4	0.008	16.0	106.8	94.9	0.000	77.3	112.4
Chhattisgarh	Korea (koriya)	118.1	0.000	61.4	174.8	72.3	0.000	54.7	89.9	84.0	0.000	62.6	105.5	80.5	0.000	64.2	96.9
Chhattisgarh	Surguja	61.4	0.097	-11.1	133.9	101.1	0.000	73.6	128.5	69.8	0.000	40.1	99.6	86.1	0.000	67.2	105.0
Chhattisgarh	Jashpur	74.6	0.100	-14.3	163.5	109.3	0.000	83.7	134.8	37.2	0.000	16.9	57.5	83.1	0.000	58.2	108.0
Chhattisgarh	Raigarh	16.3	0.408	-22.4	55.0	78.2	0.000	44.7	111.8	73.4	0.000	40.4	106.4	70.5	0.000	50.2	90.8
Chhattisgarh	Korba	79.4	0.000	43.2	115.7	101.8	0.000	81.3	122.3	71.8	0.000	50.1	93.5	85.7	0.000	70.2	101.3
Chhattisgarh	Janjgir - champa	51.4	0.001	21.2	81.5	122.8	0.001	51.5	194.1	45.9	0.000	25.7	66.2	58.0	0.000	41.4	74.5
Chhattisgarh	Bilaspur	92.7	0.000	58.4	127.1	62.3	0.000	30.4	94.2	65.6	0.000	44.0	87.1	72.0	0.000	58.1	86.0
Chhattisgarh	Kabirdham	58.2	0.004	18.6	97.8	94.1	0.000	54.2	134.1	57.5	0.000	37.6	77.5	65.7	0.000	47.3	84.1
Chhattisgarh	Rajnandgaon	85.5	0.001	33.0	138.1	49.5	0.001	21.4	77.6	71.5	0.000	48.9	94.0	66.7	0.000	47.8	85.6
Chhattisgarh	Durg	29.6	0.002	10.9	48.3	78.4	0.002	30.0	126.8	45.9	0.000	30.5	61.4	46.5	0.000	33.3	59.7
Chhattisgarh	Raipur	68.1	0.000	40.0	96.3	88.3	0.000	44.4	132.1	56.5	0.000	42.9	70.2	63.4	0.000	51.7	75.0
Chhattisgarh	Mahasamund	46.4	0.024	6.1	86.7	63.6	0.000	33.2	94.0	54.1	0.000	31.7	76.6	56.0	0.000	38.7	73.2
Chhattisgarh	Dhamtari	75.3	0.054	-1.2	151.8	79.0	0.000	45.9	112.2	58.1	0.000	32.0	84.3	66.0	0.000	47.6	84.3
Chhattisgarh	Uttar bastar kanker	23.2	0.253	-16.6	63.1	74.3	0.000	50.0	98.6	70.2	0.000	32.7	107.8	70.1	0.000	50.6	89.6
Chhattisgarh	Bastar	69.7	0.051	-0.2	139.6	105.2	0.000	80.2	130.2	72.3	0.000	38.8	105.8	95.9	0.000	77.1	114.6
Chhattisgarh	Narayanpur	57.6	0.066	-3.8	119.0	76.3	0.000	58.4	94.2	64.0	0.000	29.0	99.0	73.0	0.000	57.3	88.7
Chhattisgarh	Dakshin bastar dantewada	168.9	0.069	-13.1	350.9	99.6	0.000	72.8	126.5	130.8	0.000	88.6	173.0	106.6	0.000	88.5	124.7
Chhattisgarh	Bijapur	40.3	0.208	-22.5	103.0	58.8	0.000	41.9	75.6	86.7	0.001	35.5	137.8	61.2	0.000	46.7	75.7
Madhya Pradesh	Sheopur	91.1	0.000	54.7	127.6	141.0	0.000	98.5	183.5	56.3	0.000	38.2	74.4	84.2	0.000	64.1	104.4
Madhya Pradesh	Morena	89.8	0.000	51.6	128.1	NA	-	-	-	75.1	0.000	54.1	96.1	77.8	0.000	60.6	95.1
Madhya Pradesh	Bhind	84.6	0.000	54.0	115.3	NA	-	-	-	64.1	0.000	44.0	84.2	68.6	0.000	52.0	85.1
Madhya Pradesh	Gwalior	94.8	0.000	65.5	124.1	72.4	0.003	24.1	120.6	60.6	0.000	46.9	74.3	70.2	0.000	56.6	83.7
Madhya Pradesh	Datia	85.0	0.000	51.1	119.0	169.3	0.010	40.5	298.0	86.5	0.000	68.7	104.3	90.5	0.000	73.8	107.2
Madhya Pradesh	Shivpuri	86.5	0.000	52.2	120.7	209.8	0.000	116.7	302.9	61.2	0.000	41.0	81.4	77.3	0.000	61.3	93.3
Madhya Pradesh	Tikamgarh	92.8	0.000	51.6	134.1	180.3	0.000	101.6	259.0	75.3	0.000	55.5	95.2	89.8	0.000	69.0	110.6
Madhya Pradesh	Chhatarpur	102.7	0.000	68.4	137.0	148.9	0.001	61.8	236.1	69.9	0.000	48.5	91.3	82.9	0.000	64.2	101.7
Madhya Pradesh	Panna	142.0	0.000	86.1	197.9	129.9	0.000	78.8	180.9	107.0	0.000	83.4	130.7	117.8	0.000	97.4	138.2
Madhya Pradesh	Sagar	86.7	0.000	43.9	129.4	79.0	0.006	23.0	134.9	70.3	0.000	49.7	90.9	74.9	0.000	57.6	92.1
Madhya Pradesh	Damoh	70.3	0.000	33.0	107.6	78.6	0.006	22.5	134.6	76.8	0.000	55.3	98.4	75.6	0.000	55.1	96.2
Madhya Pradesh	Satna	120.4	0.000	69.7	171.2	121.3	0.000	65.8	176.8	40.0	0.000	22.7	57.2	70.6	0.000	56.0	85.3
Madhya Pradesh	Rewa	117.8	0.000	72.2	163.5	151.1	0.000	97.0	205.3	88.4	0.000	65.4	111.4	107.8	0.000	87.1	128.5
Madhya Pradesh	Umaria	95.6	0.001	37.9	153.3	92.5	0.000	59.4	125.6	52.2	0.000	28.0	76.4	72.2	0.000	51.6	92.9
Madhya Pradesh	Neemuch	50.9	0.053	-0.8	102.5	76.2	0.012	16.9	135.6	42.0	0.000	26.5	57.4	47.1	0.000	30.4	63.8
Madhya Pradesh	Mandsaur	58.9	0.003	20.6	97.1	44.4	0.076	-4.7	93.5	63.6	0.000	40.2	87.1	61.5	0.000	44.2	78.7
Madhya Pradesh	Ratlam	41.9	0.004	13.4	70.5	29.9	0.002	11.1	48.8	39.6	0.000	22.6	56.7	37.2	0.000	28.0	46.5
Madhya Pradesh	Ujjain	57.4	0.000	34.9	79.8	50.7	0.080	-6.1	107.5	48.7	0.000	36.3	61.2	50.8	0.000	40.7	61.0
Madhya Pradesh	Shajapur	66.5	0.000	38.5	94.4	113.9	0.074	-11.2	239.0	40.0	0.000	22.3	57.8	49.3	0.000	33.3	65.4
Madhya Pradesh	Dewas	58.9	0.001	23.2	94.6	64.1	0.000	34.7	93.6	80.0	0.000	59.6	100.4	72.8	0.000	56.1	89.5
Madhya Pradesh	Dhar	108.8	0.000	58.4	159.1	76.7	0.000	52.9	100.4	42.3	0.001	17.9	66.8	70.5	0.000	54.5	86.5
Madhya Pradesh	Indore	6.8	0.020	1.1	12.5	11.3	0.079	-1.3	23.8	29.5	0.000	17.8	41.2	21.4	0.000	13.3	29.5
Madhya Pradesh	Khargone (west nimar)	61.2	0.007	16.9	105.6	64.8	0.000	40.1	89.4	41.3	0.000	21.8	60.9	53.9	0.000	39.2	68.7
Madhya Pradesh	Barwani	50.9	0.010	12.4	89.5	54.9	0.000	38.6	71.3	58.9	0.000	30.2	87.6	55.6	0.000	43.0	68.1
Madhya Pradesh	Rajgarh	101.0	0.000	62.4	139.5	NA	-	-	-	88.5	0.000	61.8	115.3	85.7	0.000	65.6	105.8
Madhya Pradesh	Vidisha	114.2	0.000	74.0	154.4	57.1	0.008	15.1	99.2	62.9	0.000	40.5	85.3	73.6	0.000	56.4	90.9
Madhya Pradesh	Bhopal	69.1	0.000	33.4	104.8	NA	-	-	-	47.8	0.000	30.4	65.3	49.4	0.000	32.5	66.3
Madhya Pradesh	Sehore	46.4	0.018	8.0	84.8	57.2	0.022	8.3	106.2	48.9	0.000	33.8	64.0	49.8	0.000	37.9	61.7
Madhya Pradesh	Raisen	87.4	0.000	45.4	129.3	86.3	0.000	47.0	125.6	56.2	0.000	35.0	77.4	66.7	0.000	50.6	82.8
Madhya Pradesh	Betul	54.3	0.026	6.4	102.1	98.0	0.000	62.0	133.9	38.1	0.000	16.8	59.3	70.6	0.000	55.5	85.6
Madhya Pradesh	Harda	62.4	0.000	30.2	94.5	85.2	0.000	55.4	114.9	33.1	0.000	16.3	49.9	58.5	0.000	43.3	73.6
Madhya Pradesh	Hoshangabad	54.2	0.000	28.2	80.2	103.7	0.000	67.0	140.3	40.5	0.000	28.3	52.8	56.1	0.000	44.3	67.9
Madhya Pradesh	Katni	77.1	0.001	29.7	124.4	102.7	0.000	64.2	141.3	62.3	0.000	36.8	87.7	76.4	0.000	57.5	95.3
Madhya Pradesh	Jabalpur	55.3	0.000	26.3	84.3	106.3	0.000	62.6	150.0	59.4	0.000	42.7	76.0	66.5	0.000	57.4	75.6
Madhya Pradesh	Narsimhapur	45.0	0.027	5.2	84.8	79.5	0.005	23.9	135.1	35.8	0.000	17.5	54.1	45.1	0.000	29.6	60.5
Madhya Pradesh	Dindori	113.8	0.001	47.6	180.1	84.5	0.000	59.9	109.1	90.2	0.000	52.9	127.4	88.8	0.000	64.7	112.9
Madhya Pradesh	Mandla	100.3	0.044	2.8	197.9	83.4	0.000	57.5	109.3	78.0	0.000	44.9	111.2	82.2	0.000	61.5	102.9
Madhya Pradesh	Chhindwara	55.7	0.060	-2.2	113.6	86.7	0.000	51.8	121.5	52.6	0.000	34.3	70.9	65.1	0.000	46.2	84.0
Madhya Pradesh	Seoni	NA	-	-	-	74.0	0.000	45.3	102.8	27.6	0.001	10.6	44.7	46.2	0.000	30.8	61.6
Madhya Pradesh	Balaghat	114.4	0.001	47.2	181.6	81.5	0.000	36.3	126.7	66.4	0.000	46.0	86.8	73.8	0.000	53.8	93.7
Madhya Pradesh	Guna	92.7	0.000	51.3	134.2	127.6	0.000	72.0	183.3	81.2	0.000	62.7	99.7	90.1	0.000	71.8	108.4
Madhya Pradesh	Ashoknagar	58.0	0.000	27.0	89.1	58.0	0.047	0.7	115.3	39.7	0.000	23.5	55.9	46.3	0.000	33.9	58.6
Madhya Pradesh	Shahdol	94.8	0.001	36.4	153.2	70.6	0.000	37.2	104.0	75.5	0.000	45.4	105.7	76.9	0.000	57.8	96.0
Madhya Pradesh	Anuppur	51.9	0.032	4.3	99.5	88.9	0.000	60.2	117.6	41.2	0.000	21.1	61.4	68.9	0.000	54.0	83.7
Madhya Pradesh	Sidhi	139.1	0.000	90.5	187.6	75.0	0.000	38.1	111.9	82.4	0.000	59.0	105.8	90.2	0.000	72.2	108.2
Madhya Pradesh	Singrauli	55.7	0.008	14.8	96.6	91.1	0.000	63.0	119.2	74.3	0.000	52.2	96.5	79.5	0.000	65.5	93.5
Madhya Pradesh	Jhabua	128.1	0.005	39.3	217.0	58.5	0.000	45.1	71.9	18.0	0.177	-8.2	44.2	58.4	0.000	45.1	71.7
Madhya Pradesh	Alirajpur	72.3	0.000	32.8	111.9	86.7	0.000	72.3	101.0	22.2	0.197	-11.6	56.0	82.7	0.000	69.7	95.7
Madhya Pradesh	Khandwa (east nimar)	77.3	0.004	24.3	130.4	83.0	0.000	56.9	109.1	28.5	0.000	13.0	43.9	53.2	0.000	38.6	67.8
Madhya Pradesh	Burhanpur	25.0	0.196	-12.9	63.0	73.7	0.000	51.9	95.5	61.2	0.000	49.3	73.2	64.5	0.000	51.4	77.5

NA- Not applicable due to not enough sample size
