# Supplementary material for: The persistent influence of caste on under-five mortality: Factors that explain the caste-based gap in high focus Indian states
Source: PLoS One. 2019 Aug 20;14(8):e0211086. doi: 10.1371/journal.pone.0211086 (PMC6701792; doi:10.1371/journal.pone.0211086)
Supplement: S2 Table — (DOCX) [file pone.0211086.s002.docx]

**S2 Table. Neonatal mortality rate(per 1000 live births) by place of birth and ANC visit for SC,ST and Non-SC/ST population in high focus states of India, 2015-16**

| **Program indicators** | **SC** | | | **ST** | | | **Non-SC/ST** | | | **High focus states** | | | **India** | | |
| --- | --- | --- | --- | --- | --- | --- | --- | --- | --- | --- | --- | --- | --- | --- | --- |
|  | **NMR** | **95% CI** | | **NMR** | **95% CI** | | **NMR** | **95% CI** | | **NMR** | **95% CI** | | **NMR** | **95% CI** | |
|  |  | **Lower** | **Upper** |  | **Lower** | **Upper** |  | **Lower** | **Upper** |  | **Lower** | **Upper** |  | **Lower** | **Upper** |
| **Place of birth** |  |  |  |  |  |  |  |  |  |  |  |  |  |  |  |
| Home delivery | 45.8 | 40.5 | 51.2 | 39.9 | 34.8 | 45.0 | 41.8 | 38.7 | 44.9 | 42.4 | 40.3 | 44.6 | 38.5 | 36.4 | 40.5 |
| Institutional delivery | 40.5 | 37.5 | 43.5 | 33.5 | 29.5 | 37.6 | 34.0 | 32.7 | 35.4 | 35.3 | 34.1 | 36.4 | 26.0 | 25.1 | 27.0 |
| **ANC visit^$^** |  |  |  |  |  |  |  |  |  |  |  |  |  |  |  |
| None | 34.8 | 28.6 | 41.0 | 34.8 | 27.6 | 42.1 | 32.1 | 28.6 | 35.7 | 33.1 | 30.4 | 35.8 | 29.8 | 27.7 | 31.9 |
| 1 visit | 33.3 | 23.1 | 43.4 | 21.1 | 10.5 | 31.6 | 28.3 | 22.0 | 34.6 | 28.8 | 24.7 | 32.9 | 24.4 | 21.2 | 27.5 |
| 2-3 visits | 31.4 | 27.9 | 34.8 | 23.1 | 18.8 | 27.4 | 23.5 | 21.4 | 25.6 | 25.1 | 23.4 | 26.8 | 21.6 | 20.2 | 23.1 |
| 4+ visits | 26.0 | 21.0 | 31.0 | 18.6 | 14.9 | 22.3 | 17.1 | 15.3 | 18.9 | 18.8 | 17.4 | 20.1 | 13.0 | 12.1 | 14.0 |

Note: Neonatal mortality rate are estimated for five year preceding to the survey and all estimates are significant at p<0.001

^$^ANC visits during pregnancy for last birth only
