# Supplementary material for: The persistent influence of caste on under-five mortality: Factors that explain the caste-based gap in high focus Indian states
Source: PLoS One. 2019 Aug 20;14(8):e0211086. doi: 10.1371/journal.pone.0211086 (PMC6701792; doi:10.1371/journal.pone.0211086)
Supplement: S1 Appendix — (DOCX) [file pone.0211086.s003.docx]

**Appendix S1**

**Description of Fairlie method**

Since the independent contribution of each variable in the non-linear decomposition depends on the order in which the variables are introduced in the model, we randomised the order of the variables as suggested by Fairlie (2005) [29] to get robust estimates. For the decomposition analysis, we combine the social group predictor SCs and STs and refer to them as ‘SC/ST’ and the rest of the population is referred to as the ‘non-SC/ST’.

The decomposition method proposed by Fairlie is described below. As per the Standard Blinder-Oaxaca decomposition, the SC/ST vs non-SC/ST gap in the average value of the dependent variable, Y, (here, under-five mortality) can be expressed as

, (i)

where, $\bar{X}^{j}$ is a row vector of average values of the independent covariates and $\hat{\beta}^{j}$is a vector of coefficient estimates for under-five mortality j. An extension of this decomposition for a non-linear equation, Y= F ($X\hat{\beta}$), can be written as

, (ii)

An equally valid expression for the decomposition is:

, (iii)

Where, $N^{j}$is the sample size for interest group j. $Y^{j}$is the average probability of the binary outcome of the interest group *j* and *F* is the cumulative distribution function from the logistic distribution. Here, superscripts ‘n’ and ‘s’ stand for ‘non-SC/ST’ and ‘SCs/STs’ population.

In both (ii) and (iii), the first term in brackets represents the part of the gap between social groups due to group differences in distributions of the entire set of independent variables, and the second term represents the part due to differences in the group processes determining the levels of Y. The second term also captures the portion of the group gap due to group differences in unmeasurable or unobserved endowments.

For total contribution, we need to calculate two sets of predicted probabilities for SCs/STs and the non-SC/ST population and take the difference between the average values of the two. However, obtaining the contribution of a specific covariate is not direct. As the sample sizes of the two groups are not the same, we need to carry out a regression for pooled data (SCs/STs and the non-SC/ST population together) and calculate the predicted probabilities, for each SCs/STs and the non-SC/ST population observation in the sample. Since the non-SC/ST population is bigger than SCs/STs, a random subsample of the non-SC/ST population equal in size to the full SCs/STs sample should be drawn. Each observation in the non-SC/ST population sample and full SCs/STs sample is then separately ranked by predicted probabilities and matched by their respective rankings. This procedure matches the SCs/STs under-fives who have characteristics placing them at the bottom (top) of their distribution with under-fives from the non-SC/ST population who have characteristics placing them at the bottom (top) of their distribution. Now assume that N_s_=N_n_ and a natural one-to-one matching of SCs/STs and non-SC/ST population observations exists. Also, assume that there are two independent variables to explain the social group gap in under-five mortality.

Using coefficient estimates from a logit regression for a pooled sample,$\hat{\beta}^{*}$, the independent contribution of X_1_ to the group gap can then be expressed as:

Similarly, the contribution of X_2_ can be expressed as:

The contribution of each variable to the gap is thus equal to the change in the average predicted probability from replacing the SCs/STs distribution with the non-SC/ST population distribution while holding the distributions of the other variables constant. However, the assumption of equal sample size is rarely true in practical situations. Since the non-SC/ST sample is substantially larger, a large number of random subsamples of the under-five of the non-SCs/STs (equal size to total SCs/STs sample) are drawn to match each of them to the SCs/STs sample and calculate separate decomposition. Finally, the mean value of all these separate decomposition estimates is used as an approximate decomposition for the entire non-SC/ST population sample. We used 1000 replications of such decompositions and presented the average result. It must be noted here that increasing the number of replications improves the stability of the results.

**Sensitivity analysis**

Table1 shows the sensitivity analysis of the Fairlie decomposition estimates of different covariates. The decomposition estimates obtained from this procedure depend on the randomly chosen order of the covariates. There is no substantial change in the results once we change the order of the variables.

**Table1. Sensitivity analysis of the Fairlie decomposition estimates of different covariates**

| **Covariates** | **Contribution coefficient in percentages** | **Covariates** | **Contribution coefficient in percentages** |
| --- | --- | --- | --- |
| Mother’s age at first birth | -3% | Sex of the child | -4% |
| Type of fuel used for cooking | 10% | Place of delivery | 8% |
| Type of toilet | -3% | Type of fuel used for cooking | 10% |
| Source of drinking water | -8% | Type of toilet | -3% |
| Sex of the child | -4% | Source of drinking water | -7% |
| Place of delivery | 9% | Mother’s age at first birth | -3% |
| Birth order | -2% | ANC visit | 6% |
| Wealth of the household | 68% | Type of residence | 5% |
| Mother’s level of education | 18% | Birth order | -2% |
| Type of residence | 4% | Wealth of the household | 71% |
| ANC visit | 11% | Mother’s level of education | 19% |
